# Dynamic evolution and hierarchical regulation of rDNA in polyploid oat

**DOI:** 10.1111/tpj.71140

**Published:** 2026-09-22

**Authors:** Ziyang Wan, Haorang Wang, Jie Qin, Wentao Ding, Xin Wang, Qianqian Ge, Handong Su

**Affiliations:** ^1^ National Key Laboratory of Crop Genetic Improvement, Hubei Hongshan Laboratory Huazhong Agricultural University Wuhan 430070 China

**Keywords:** *Avena*, ribosomal DNA (rDNA), polyploidy, rRNA transcription, nucleolar dominance

## Abstract

Ribosomal DNA (rDNA) encodes the RNA core of ribosomes, yet it remains one of the least understood components of eukaryotic genomes, particularly in complex polyploids. Here, we present a comprehensive analysis of rDNA organization, evolution, and transcription across the *Avena* genus. We show that rDNA copy number varies extensively across loci and genotypes, and is partially structured by populations. Despite widespread sequence polymorphism, rRNA coding regions exhibit greater sequence conservation than noncoding regions, which retain considerably high levels of sequence diversity. Comparative analyses across ploidy levels further reveal that rDNA evolution is highly structured, lineage‐specific, and asymmetric, with polyploidization accompanied by extensive remodeling of rDNA composition and preferential retention of particular subgenome‐derived variants. At the transcriptional level, rDNA arrays are not used uniformly, but instead show context‐dependent expression of specific variants across developmental and environmental conditions. Using oat–maize addition lines, we further demonstrate complete silencing of alien maize rDNA, while endogenous oat rDNA preserves a stable internal transcriptional hierarchy. Together, these findings establish rDNA as a dynamic genomic system shaped by genome merger, selection, and hierarchical regulation during polyploid evolution. More broadly, they provide a framework for understanding how ribosomal gene diversity contributes to regulatory plasticity, genome stability, and cellular robustness in complex crop genomes.

## Significance Statement

How ribosomal DNA evolves and functions following polyploidization remains a fundamental question in plant genome biology. This study uncovers extensive remodeling of rDNA dosage, sequence composition, and transcription across the oat genus, providing new insights into ribosomal gene evolution and nucleolar regulation in polyploid plants.

## INTRODUCTION

Ribosomes are the universal engines of cellular growth and homeostasis, catalyzing protein synthesis in all living organisms. Their structural and catalytic core consists of ribosomal RNAs (rRNAs), which are encoded by ribosomal DNA (rDNA) arranged as long tandem arrays within nucleolar organizing regions (NORs) (Hori et al., 2023). A typical 45S rDNA repeat contains the 18S, 5.8S, and 25–28S rRNA genes, separated by internal transcribed spacers (ITS) and flanked by external transcribed spacers (ETS), while adjacent repeats are divided by an intergenic spacer (IGS) (Sáez‐Vásquez & Delseny, 2019). Despite their indispensable roles, rDNA loci remain among the most poorly characterized regions of eukaryotic genomes because their extreme repetitiveness, structural complexity, and sequence homogeneity collectively hinder genome assembly and functional analysis (Li, Wang, et al., 2025; Liu, Li, et al., 2025).

This technical barrier has obscured an emerging view of rDNA as a dynamic and heterogeneous genomic system. Rather than being strictly uniform and rapidly homogenized, recent studies have revealed that rDNA arrays can vary extensively in copy number, sequence composition, and chromatin state. Across eukaryotes, rDNA copy number ranges from tens to thousands and can differ substantially even within a species (Nelson et al., 2019). In yeast and mammals, such variation has been linked to altered transcription output, cancer susceptibility, and aging (Gibbons et al., 2014; Hotz et al., 2022; Law et al., 2024; Rodriguez‐Algarra et al., 2024). In plants, rDNA dosage and expression have also been associated with key agronomic traits, including flowering time and stress responsiveness (Lee et al., 2024; Li et al., 2018). In parallel, population‐scale analyses and telomere‐to‐telomere assemblies have uncovered widespread sequence variation within rDNA arrays, including single‐nucleotide polymorphisms (SNPs), short insertions and deletions (Indels), structural rearrangements, transposon element insertions, and diverse epigenetic states (Chen et al., 2023; Fultz et al., 2023; Hall et al., 2022; Hori et al., 2021). Together, these findings challenge the classical model of strict concerted evolution and instead suggest that rDNA is shaped by multiple, uneven selective pressures acting across functional levels (Parks et al., 2018; Sultanov & Hochwagen, 2022). rDNA arrays may therefore contribute not only to ribosome biogenesis, but also to regulatory flexibility and lineage‐specific adaptation (Genuth & Barna, 2018; Kurylo et al., 2018; Preiss, 2016; Rothschild et al., 2024; Shi et al., 2017; Sims et al., 2021; Simsek et al., 2017; Welfer et al., 2025).

These questions are especially relevant in allopolyploids, where hybridization and whole‐genome doubling bring together divergent parental rDNA arrays within a common nucleus (Leitch & Leitch, 2008; Otto, 2007). One of the most striking consequences is nucleolar dominance (ND), in which rDNA inherited from one parental genome is preferentially transcribed while the other is silenced (Borowska‐Zuchowska et al., 2020; Navashin, 1934; Tucker et al., 2010). ND has been documented in diverse plant allopolyploids and is associated with DNA methylation, histone modification, and large‐scale chromatin reorganization (Guo & Han, 2014; Rabanal et al., 2017). In wheat, for example, rDNA loci from the A and D subgenomes are often silenced or eliminated, whereas those from the B (S) subgenome remain transcriptionally dominant in both natural and synthetic allopolyploids (Guo & Han, 2014; Handa et al., 2018). These asymmetries can arise rapidly after hybridization and recur independently across polyploidization events, underscoring their evolutionary significance (Borowska‐Zuchowska et al., 2023). Yet a central question remains unresolved: how polyploidization reshapes rDNA copy number, sequence diversity, and transcriptional output, and how these processes interact during genome stabilization.

The genus *Avena* provides an excellent system to address this question (Clemens & van Klinken, 2014). Common oat (*A. sativa* L., 2n = 6x = 42, AACCDD) is a globally important cereal crop, and the genus includes approximately 30 diploid, tetraploid, and hexaploid species with diverse genome compositions (Avni et al., 2025; Zhang et al., 2025). Hexaploid oat originated through hybridization between a diploid ancestor (*A. longiglumis*, 2n = 2x =14, AlAl) and a tetraploid species (*A. insularis*, 2n = 4x = 28, CCDD), followed by genome doubling (Kamal et al., 2022; Peng et al., 2022). Cultivated oat includes both hulled and naked forms, and recent genomic evidence suggests an independent domestication history for naked oat (Nan et al., 2023). High‐quality pangenomic and transcriptomic resources now make it possible to examine repetitive genome components in this system with unprecedented resolution (Avni et al., 2025; Li, Wang, et al., 2025; Zhang et al., 2025). A recent study has further revealed that polyploidization has profoundly reshaped genome architecture in oat, including the evolution of non–B‐form DNA structures across centromeres of subgenomes (Liu et al., 2023), highlighting the extensive genomic remodeling that accompanies allopolyploid evolution. In addition, early distant hybridization between oat and maize generated oat‐maize addition (OMA) lines, each carrying a single pair of maize chromosomes in an otherwise intact oat background (Kynast et al., 2001; Riera‐Lizarazu et al., 1996). These lines offer a unique and simplified experimental system for testing how heterologous chromosomes influence rDNA regulation in an interspecific genomic context (Dong et al., 2018; Wang et al., 2014).

Here, we combine cytogenetic, genomic, population‐scale, and transcriptomic analyses to define the organization, evolution, and transcriptional regulation of rDNA across the genus *Avena*. Our results show that hybridization and genome doubling events are accompanied by remodeling of rDNA arrays, leading to a highly structured and asymmetric manner of sequence variation and transcription. Together, these results establish rDNA as a dynamic genomic system shaped by genome merger, selection, and regulatory hierarchy during polyploid evolution.

## RESULTS

### rDNA copy number is highly variable and population‐stratified in hexaploid oat

**Figure 1:**
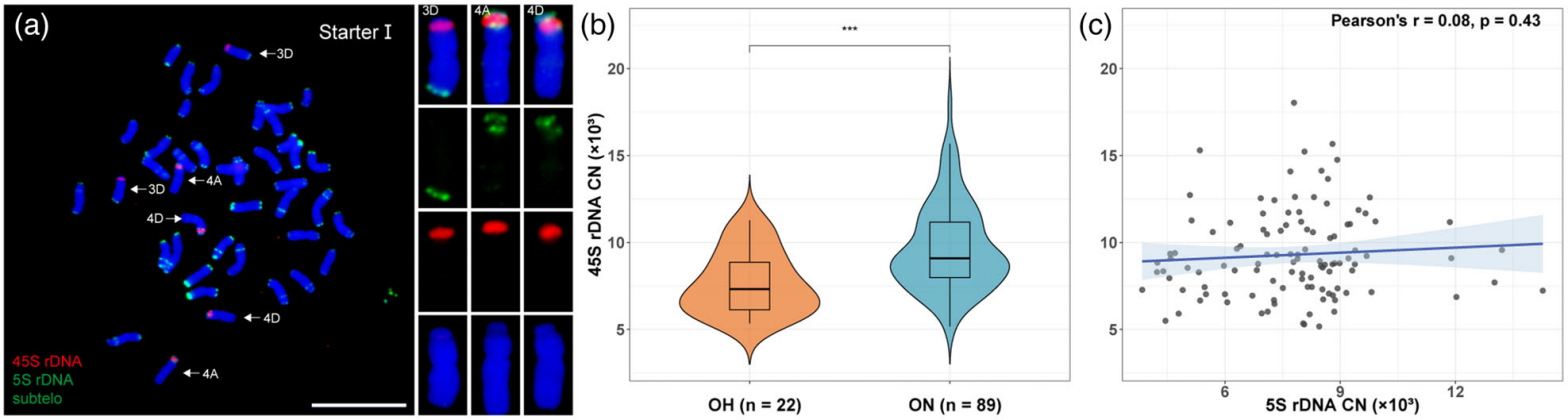
Chromosomal distribution and copy number variation of rDNA in hexaploid oat. (a) Fluorescence in situ hybridization (FISH) mapping of rDNA loci in hexaploid oat (cv. *Starter I*). 45S (red), 5S rDNA (green) and subtelo (green) signals are visualized on mitotic metaphase chromosomes. 45S rDNA loci were localized to the terminal regions of the short arms of chromosomes 3D, 4A, and 4D. Insets show enlarged views of representative chromosomes. Chromosomes are counterstained with DAPI (blue). Scale bars: 20 μm. (b) Violin plots showing the comparison of 45S rDNA copy number in hulled oat (OH, *n* = 22) and naked oat (ON, *n* = 89). Wilcoxon rank‐sum test, ***P < 0.001. (c) Scatter plot showing the relationship between 5S and 45S rDNA copy number across accessions. Linear regression (blue line) reveals weak correlation (r = 0.08, *P* = 0.43), indicating largely independent regulation of two rDNA classes in oat.

To examine the chromosomal localization of rDNA in hexaploid oat, we performed fluorescence in situ hybridization (FISH) using 45S and 5S rDNA probes in two cultivars (*Starter I* and *Sun II*). In both genotypes, three pairs of NORs were consistently detected on the short arms of chromosomes 3D, 4A, and 4D (Figure 1a, Figure S1a). No 45S rDNA signals were detected on C‐genome chromosomes, indicating that C‐genome rDNA loci were lost during the first polyploidization step of oat evolution. Although NOR positions were conserved, 45S signal intensity varied substantially among chromosomes and between cultivars (Figure 1a, Figure S1a). This heterogeneity indicates pronounced locus‐specific and genotype‐dependent differences in rDNA copy number.

We then quantified this variation using the normalized whole‐genome sequencing depth method (Li et al., 2018) across diploid, tetraploid, and hexaploid *Avena* species (Table S1). Background read depth (BRD) estimates based on single‐copy exons and introns were highly consistent (Pearson's r = 0.98, *P* = 7.76 × 10^−12^; Figure S1b,c), and saturation analysis showed that sequencing depths above 5× were sufficient for reliable copy number estimates (Figure S2). To independently validate these estimates, we performed qPCR on representative accessions. The qPCR‐derived copy‐number estimates were consistent with those obtained from sequencing data (Table S2), supporting the reliability of the read‐depth‐based approach. Across ploidy levels, 45S rDNA copy number increased stepwise, from ~3638 in diploids to ~6165 in tetraploids and ~ 13 546 in hexaploids (Figure S3), indicating substantial retention of rDNA arrays through successive polyploidization events rather than simple dosage loss.

We next assess population‐level variation using 111 hexaploid accessions, including 22 hulled (OH) and 89 naked oat (ON) (Nan et al., 2023). rDNA copy number ranged nearly two orders of magnitude, from 5172 to 18 036 copies per genome (Table S3), revealing substantial variation among accessions. Moderate population differentiation was detected (V_st_ = 0.116), with naked oat tending to harbor higher copy numbers than hulled oat (Figure 1b), indicating population‐level differences in rDNA dosage. This pattern may reflect distinct evolutionary processes, including demographic history, population structure, unequal inheritance, domestication, or other historical factors. In contrast, 45S and 5S rDNA copy numbers were only weakly correlated (Pearson's r = 0.08; Figure 1c), and similarly weak relationships were observed between 5S rDNA and the individual 18S, 5.8S, and 28S components of the 45S unit (Figure S4), indicating that these repeat families are regulated largely independently. Together, these results showed that rDNA dosage in oat is highly dynamic across evolutionary and population scales.

### Extensive sequence polymorphism reveals contrasting patterns of sequence conservation across oat rDNA

**Figure 2:**
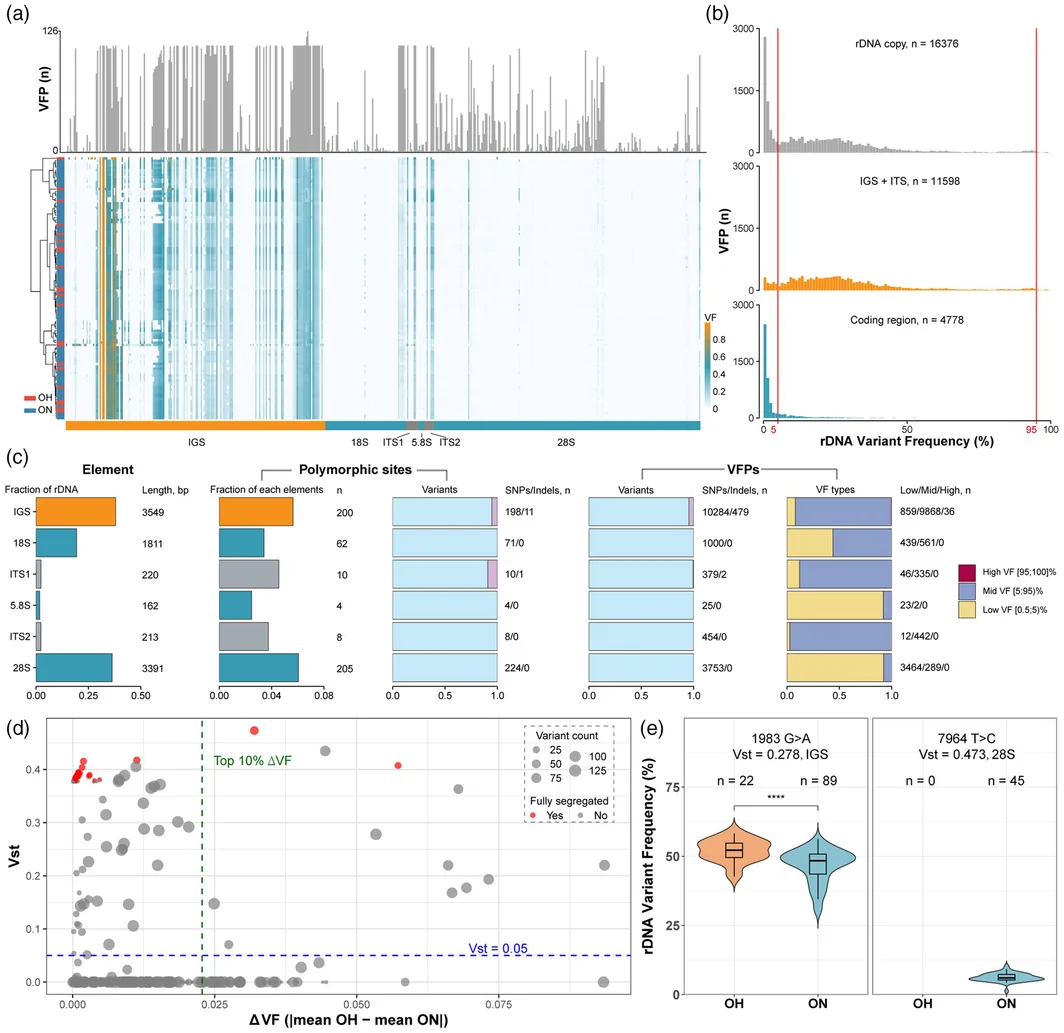
Sequence polymorphism and population differentiation of rDNA in oat. (a) Genome‐wide landscape of rDNA sequence variation across the oat population. The top histogram shows the distribution of rDNA variant frequency polymorphisms (VFPs) along the full‐length rDNA unit. The middle heatmap displays variant frequency (VF) across accessions (OH, red; ON, blue). The bottom panel shows rDNA structural annotation. (b) Histograms showing VF distributions for variants across the full‐length rDNA unit (top), noncoding (IGS + ITS; middle), and coding regions (bottom). Red vertical lines indicate thresholds for low‐frequency (< 5%) and high‐frequency (> 95%) variants. (c) Bar plots summarizing the distribution of sequence variation across rDNA components. Element indicates the relative proportion and absolute length of each component within the rDNA unit. Polymorphic sites show the total variant counts in each region, together with the relative proportions of SNPs and Indels. VFPs compare the distribution of variants across low‐, mid‐, and high‐frequency classes. (d) Scatter plot showing the population differentiation of rDNA variants, indicating by distribution of population differentiation index (Vst) and VF difference (ΔVF) between OH and ON populations. Each point represents a polymorphic site with point size proportional to variant counts. Dashed lines indicate the Vst threshold (blue, 0.05) and top ΔVF percentile (green, 10%). Red dots denote fully segregated variants. (e) Violin plots showing rDNA variant frequency distributions of representative divergent variants in IGS (G1983A) and 28S (T7964C) (****P < 0.0001).

We next asked how much sequence diversity is embedded within oat rDNA arrays. We define rDNA variant frequency (VF) as the proportion of sequencing reads supporting a given variant among reads mapped to the corresponding position within the multicopy rDNA repeats. Across the same 111 hexaploid accessions, LoFreq analysis (Wilm et al., 2012) identified 16 376 rDNA variant frequency polymorphisms (VFPs) corresponding to 527 distinct variants across 489 polymorphic sites (Figure 2a; Table S4). Across the polymorphic sites, most rDNA variants were single‐nucleotide substitutions or short indels (1–21 bp) (Figure S5a). 65.8% (*n* = 332) were detected in fewer than 30 accessions, whereas only 13.1% (*n* = 64) were nearly ubiquitous (Figure S5b). Two relatively high‐frequency variants (G3204A 12.0–46.3%; C5460T 13.1–50.2%) were independently validated by cleaved amplified polymorphic sequence (CAPS) assays (Figure S6). These data showed that oat rDNA arrays harbor extensive variation both within genomes and among accessions.

These sequence variations were distributed unevenly across the rDNA repeat. At the population‐level, 70.8% of polymorphic variant sites were located in noncoding regions (IGS + ITS) (Figure 2b). Each accession carried, on average, 147.5 ± 25.7 variants (median = 144; range = 109–225), with significantly greater diversity in noncoding (104.5 ± 6.4) than coding regions (43.0 ± 21.8) (Figure S7a). Consistent with these observations, sliding‐window analyses revealed substantially lower repeat‐copy nucleotide diversity and variant density across the 18S, 5.8S, and most of the 28S rRNA coding regions than across the IGS, ETS, and ITS regions (Figure S7b,c). Variant number was not correlated with rDNA copy number (Pearson's r = −0.17), indicating that increased rDNA dosage does not simply generate increased sequence diversity (Figure S7d). rDNA variant frequency distributions further reinforced this contrast across functional regions of the rDNA repeat: noncoding variants spanned a broader frequency range (5–95%), whereas coding‐region variants were overwhelmingly rare (VF < 5%) (Figure 2c). High‐frequency variants (VF > 95%) were uncommon overall and were largely confined to the IGS (*n* = 36). These patterns indicate markedly different levels of sequence conservation across the rDNA repeat. Coding regions remain substantially more‐conserved than noncoding regions, consistent with strong evolutionary constraint on rRNA genes.

Although hulled and naked oat showed broadly similar overall rDNA variant counts and frequency spectra (Figure S8), 40 polymorphic sites showed clear population differentiation, enriched in the IGS (*n* = 13) and 28S (*n* = 19) regions (Figure 2d,e; Table S5). Coding‐region variants with high differentiation typically remained at low frequencies (0–10%), yet still showed reproducible divergence between populations (Figure S9). Notably, 30 variants were nearly absent in the hulled group but present in the naked group, and the strongest differentiation variant was observed at T7964C in the 28S gene (Figure 2e). In addition, we identified 19 pairs of co‐varying variants, including 8 within coding regions (Figure S10). Most coupled variants exhibited high intragenomic variant fractions, indicating co‐occurrence within the same rDNA copies. Collectively, these results reveal abundant sequence variation within oat rDNA arrays together with pronounced differences in the distribution of sequence variants across functional regions and among populations, providing a foundation for investigating the evolutionary processes shaping rDNA diversity.

### rDNA variants follow distinct evolutionary trajectories across the *Avena* genus

To determine how rDNA evolves across the genus, we profiled 59 representative accessions spanning diploid, tetraploid, and hexaploid species and ten genome types (Ac, Ad, Al, As, Cp, Cv, AB, AcAs, CD, and ACD). Across accessions, we detected 72–165 polymorphic sites per genome, with substantial differences among genome types (Figure S11a; Table S6). In most genomes, more than 90% of variations were concentrated in the IGS. By contrast, C‐lineages (Cp and Cv) showed a striking redistribution of variation into ITS and 28S regions (Figure S11b), indicating that rDNA sequence evolution in *Avena* follows lineage‐specific trajectories rather than a common pattern.

**Figure 3:**
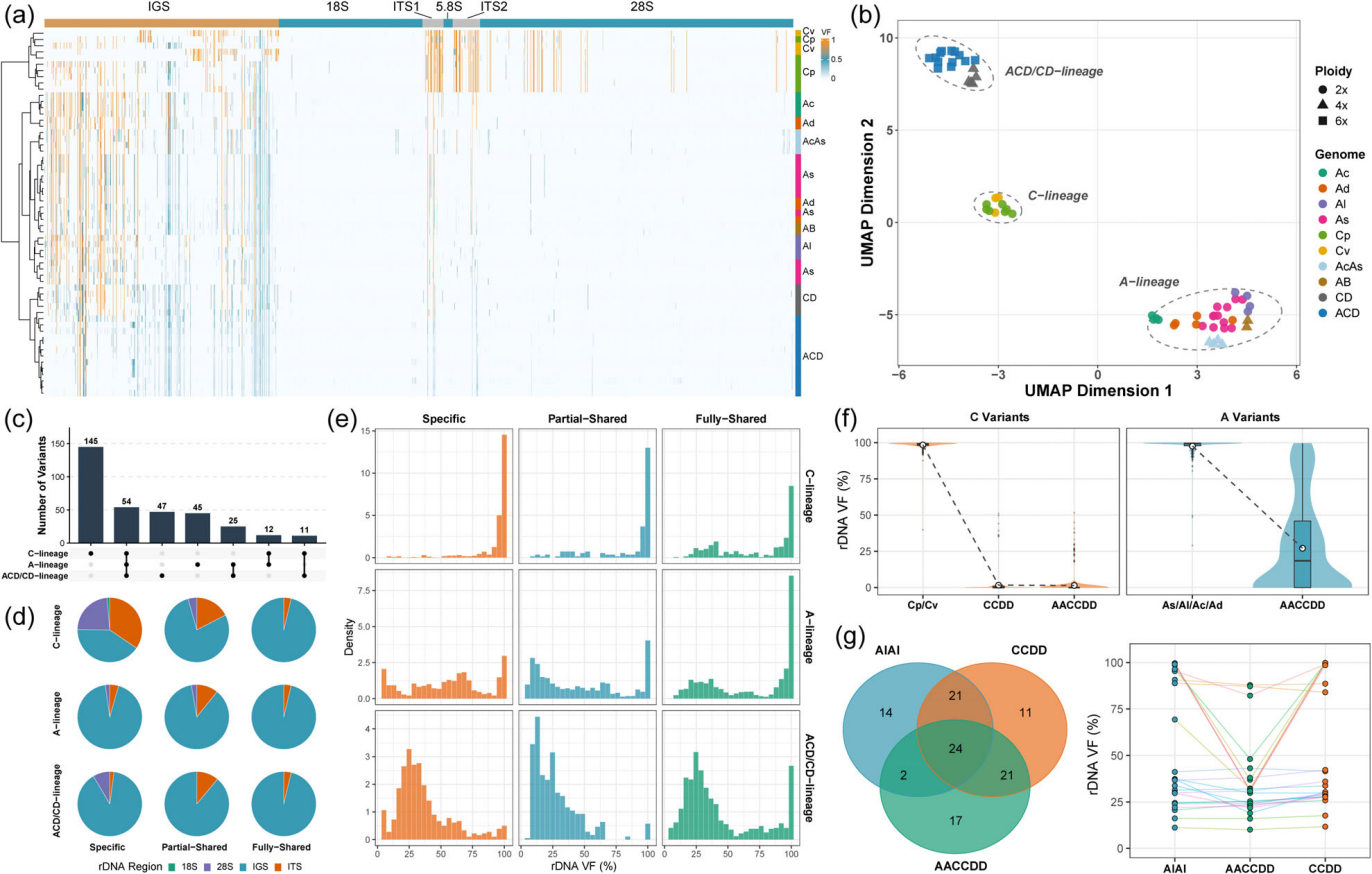
Lineage‐specific and asymmetric evolution of rDNA across the genus *Avena*. (a, b) Hierarchical clustering based on VF matrices (a) and UMAP projection (b) of rDNA variants across 59 *Avena* accessions. Rows represent accessions and columns represent polymorphic sites. Colors indicate VF from 0 to 1. Top annotation indicates rDNA structural regions, and side annotation indicates genome types. (c) Numbers of lineage‐specific, partially‐shared, and fully‐shared rDNA variants across three lineages. UpSet plot summarizes the overlap structure of polymorphic sites. (d) Distribution of lineage‐specific, partially‐shared, and fully‐shared variants across rDNA structural regions. Pie charts show the relative contributions of IGS, ITS, 28S, and 18S regions within each variant class and lineage. (e) rDNA variant frequency distributions of lineage‐specific, partially‐shared, and fully‐shared variants across different lineages. (f) Frequency dynamics of subgenome‐specific variants during polyploidization. Left: C‐lineage specific variants across diploid Cp/Cv, tetraploid CD, and hexaploid ACD genomes. Right: A‐genome–specific variants across diploid A and hexaploid ACD. Dashed lines connect rDNA variant frequency dynamics across ploidy levels. (g) Venn diagram showing the overlap of polymorphic sites among diploid Al, tetraploid CD, and hexaploid ACD genomes. Frequency trajectories of the 24 shared variants across the three ploidy levels, illustrating both conserved and attenuated evolutionary patterns.

To assess whether this variation reflects a phylogenetic relationship, we performed hierarchical clustering and UMAP dimensionality reduction assays based on rDNA variant frequency matrices. Both analyses resolved three sharply defined groups corresponding to C‐lineage, A‐lineage, and ACD/CD‐lineage (Figure 3a,b). Although the IGS contained most polymorphisms, it contributed little to lineage separation (*n* = 10), whereas ITS (*n* = 34) and 28S (*n* = 21) variants were strongly lineage‐informative (Figure S11c,d). Thus, the most phylogenetic informative rDNA variants are not the most abundant but those that are most strongly associated with lineage divergence. Overall, rDNA variation exhibits a highly structured distribution pattern that closely mirrors genome composition and evolutionary relationships across *Avena* (Bai et al., 2025; Zhang et al., 2025).

Lineage‐specific analyses further revealed pronounced asymmetry of rDNA variation (Figure 3c, Figure S12). The C‐lineage carried many more private variants (*n* = 145) than either the A‐ (*n* = 45) or ACD/CD‐lineages (*n* = 47). In contrast, only 54 rDNA variants were shared across all three lineages (Figure 3c), likely representing a conserved rDNA core. Notably, lineage‐specific variants in the A‐ and ACD/CD‐lineages remained largely confined to the IGS, whereas in the C‐lineage they extended into ITS and 28S regions (Figure 3d). This pattern was consistent with the elevated coding‐region polymorphism observed in the diploid C‐genome and further indicated that rDNA evolution has followed distinct trajectories among *Avena* lineages.

rDNA variant frequency spectra also differed markedly among lineages (Figure 3e). Many C‐lineage‐specific variants occurred at high frequencies within detectable rDNA repeats, consistent with relatively low intragenomic sequence heterogeneity. The A‐lineage retained a much broader range of rDNA variant frequencies, consistent with lower levels of sequence homogenization. By contrast, the ACD/CD‐lineage was enriched for intermediate‐frequency variants and contained fewer high‐frequency sites, a pattern consistent with incomplete homogenization of rDNA repeats after polyploidization (Figure 3e). Shared variants tended to occur at higher frequencies, whereas lineage‐specific variants were much more heterogeneous (Figure 3e, Figure S12). Together, these results reveal distinct lineage‐associated frequency distributions and indicate that the extent of rDNA homogenization differs among *Avena* lineages.

We then traced the fate of genome‐specific variants during polyploidization. Using stringent criteria (VF > 90% in source lineage and absent elsewhere), we identified 54 C‐lineage and 17 A‐lineage specific rDNA variants (Figure S13; Table S7). C‐genome variants were rapidly purged after formation of tetraploid CCDD (median VF ≈ 0; prevalence 2.96%) and were nearly absent in hexaploids. In contrast, A‐genome variants, although reduced in frequency (median VF 18.5%), remained broadly retained in hexaploid oat (Figure 3f). This contrasting pattern indicates extensive and asymmetric remodeling of parental rDNA sequence composition following polyploidization, characterized by greater retention of A‐subgenome‐specific variants and a marked reduction of C‐subgenome‐specific variants. The molecular mechanisms underlying this asymmetry cannot be resolved from the present data.

Despite this extensive turnover, a subset of variants remained stable across ploidy levels (Figure 3g, Figure S14). Most of these conserved variants persisted at high frequency from diploids to hexaploids (*n* = 18), defining a stable core of the rDNA array, whereas a small number declined progressively (e.g., C3008T and T3054C), indicating dilution or selective loss during polyploid evolution. Together, these results demonstrate that rDNA variation in *Avena* is highly structured, lineage‐specific, and asymmetrically remodeled following polyploidization, while a subset of variants remains conserved across ploidy levels.

### rRNA variants show dynamic, context‐dependent expression in hexaploid oat

**Figure 4:**
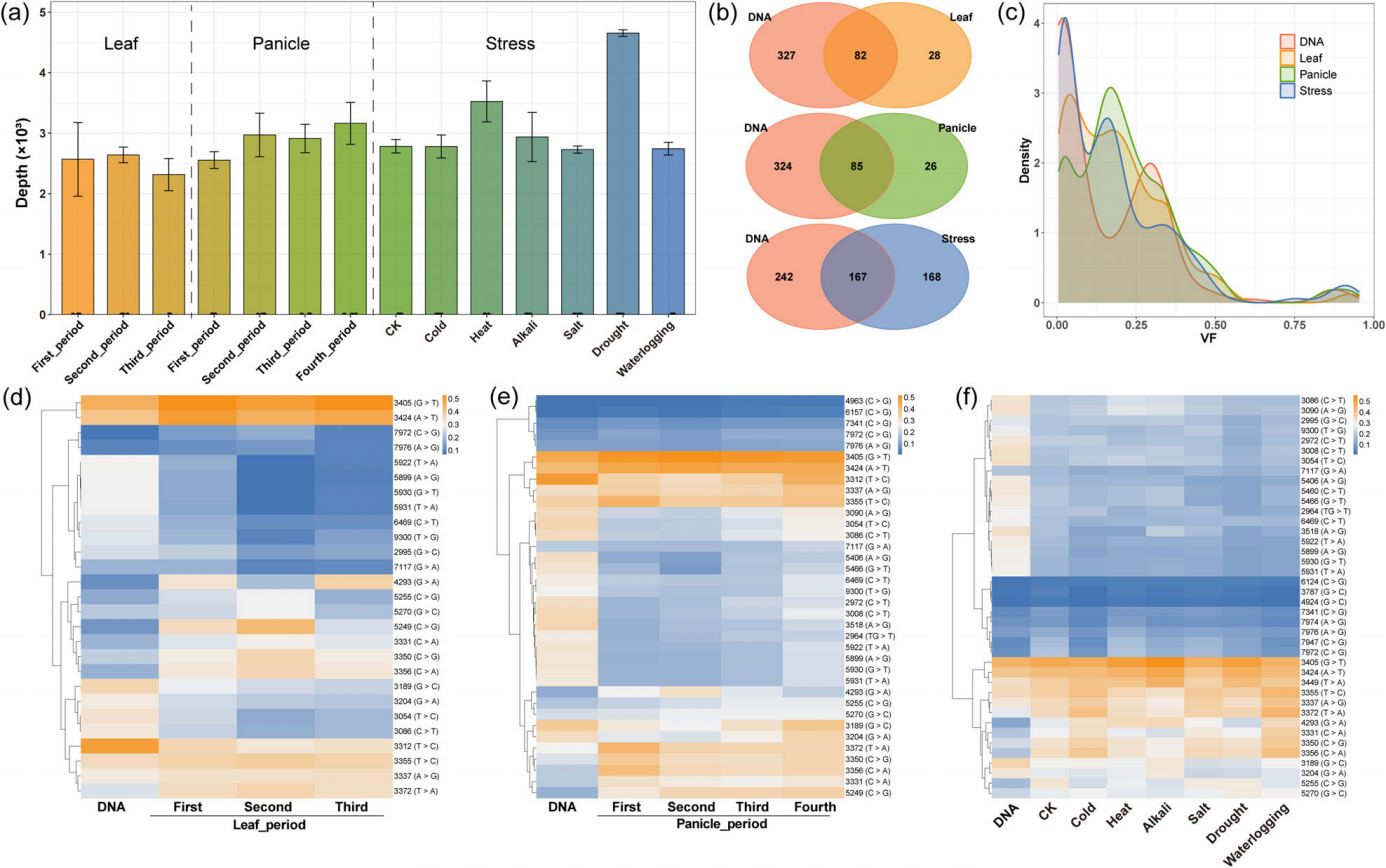
Transcriptional dynamics of rDNA variants across development and stress conditions in hexaploid oat. (a) Sequencing depth across the 45S rDNA unit in leaf, panicle, and stress‐treated. Error bars indicate standard deviation. (b) Venn diagrams showing partial overlap between variants detected in genomic DNA and those observed in transcriptomes. (c) Density plots showing shifts in rDNA variant frequency between genomic and transcriptomic datasets. (d–f) Heatmaps displaying rDNA variant frequencies of representative rDNA variants, with genomic DNA as baseline and RNA profiles (d, leaf development; e, panicle development; f, abiotic stress treatments) representing transcriptional output. Variant positions and nucleotide substitutions are indicated on the right.

We next asked whether genomic rDNA variation is translated directly into transcriptional output. To address this, we analyzed 43 RNA‐seq libraries from hexaploid oat spanning multiple tissues, developmental stages, and abiotic stress conditions (Table S8). Across all samples, sequencing depth over the 45S rDNA unit was consistently high (approximately 2 × 10^3^–5 × 10^3^) and reproducible, with strongest coverage in the 18S, 5.8S, and 28S regions (Figure 4a, Figure S15). These data provided a reliable framework for resolving expressed rDNA variants across biological contexts.

Using a stringent consensus‐based approach, we identified a core set of transcribed variants that largely overlapped with genomic polymorphisms (Figure 4b, Figure S16b). Notably, the number of expressed variants increased under stress (167 in stress conditions vs. 82 in leaf and 85 in panicle), indicating that rDNA transcriptional diversity expands under environmental challenge. RNA‐specific variants were rare and declined with increasing sequencing depth (Figure S16c), indicating that most detected transcriptional variations reflect unequal use of existing rDNA copies rather than technical noise. Thus, a substantial fraction of genomic rDNA diversity is transcriptionally engaged. We next asked whether these rDNA variants contribute equally to the rRNA pool. Across all conditions, RNA‐level variant frequency distributions were shifted toward lower values relative to genomic DNA (Figure 4c), revealing that only a subset of rDNA copies dominates transcription. Variants that were common at the DNA level were often underrepresented in RNA, indicating that transcription is not proportional to copy number but may be selectively biased among rDNA units.

This bias was also highly dynamic. Tracking variant frequencies across development, tissues, and stress treatments revealed broad and coordinated shifts in variant abundance, indicating active remodeling of rDNA transcription (Figure 4d,f). A small subset of variants remained stable across all conditions (e.g., G3405T and A3424T in IGS), marking constitutively active rDNA units, whereas most variants showed strong context dependence. Developmental transitions were accompanied by clear shifts in variant representation, and reproductive tissues displayed distinct transcriptional profiles relative to vegetative tissues. For instance, variants in the 18S region (G4293A and C5249G) exhibited strong stage‐specific fluctuations in rDNA variant frequency during leaf development (Figure 4d). Stress further amplified this plasticity: some variants remained consistently repressed (e.g., C6124G, G3787C, and G4924C), whereas others were activated in a treatment‐specific manner (Figure 4f). For instance, the G4293A variant showed increased frequencies under multiple stresses except drought, whereas the G5270C variant was specifically upregulated under drought conditions. Notably, distinct stresses therefore engaged distinct subsets of rDNA variants rather than eliciting a uniform response. Together, these results showed that rDNA in hexaploid oat does not behave as a uniform multi‐copy array, but as a regulated repertoire whose usage is selectively modulated across developmental and environmental contexts.

### Nucleolar dominance and variant‐level tuning shape rDNA expression in oat–maize addition lines

**Figure 5:**
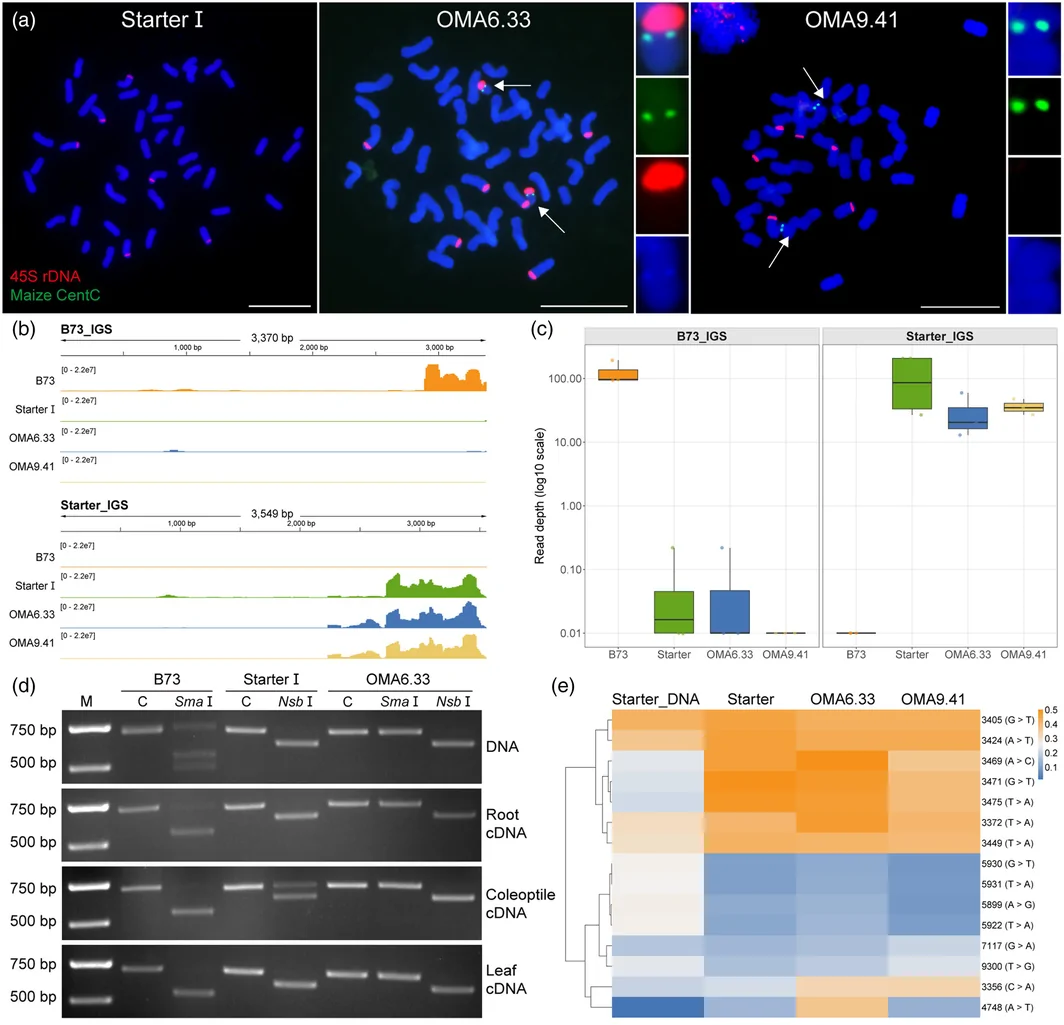
Cytological and molecular characterization of rDNA in oat–maize addition lines. (a) FISH detection of rDNA loci in oat–maize addition lines using 45S rDNA (red) and maize centromeric repeat CentC (green). Arrows indicate alien maize chromosomes. Chromosomes are counterstained with DAPI (blue). Scale bars, 20 μm. (b) Integrative Genomics Viewer (IGV) snapshots showing RNA‐seq coverage across oat and maize IGS regions. The y‐axis scale is consistent across all samples. (c) Quantification of normalized sequencing depth (log10 scale) over species‐specific IGS regions. (d) RT‐CAPS assays of rRNA gene expression in OMA6.33 line. Each sample includes genomic DNA template (the first lane) and RNA template (cDNA, the second lane). Restriction digestion (*Sma* I and *Nsb* I) of PCR amplicons confirms representative SNPs identified from sequencing in parental and OMA6.33 lines. (e) Heatmap of rDNA variant frequencies for representative shared rDNA variants comparing genomic DNA in the oat parent and RNA from OMA lines.

To test how a heterologous chromosomal environment affects rDNA organization and expression, we analyzed two oat–maize addition lines, OMA6.33 and OMA9.41 (Dong et al., 2018). OMA6.33 carries maize chromosome 6, which contains a 45S rDNA locus, whereas OMA9.41 carries maize chromosome 9, which lacks rDNA. This design allowed us to distinguish the effects of alien chromosome presence from those of alien rDNA itself. Cytological analyses showed that the endogenous oat rDNA loci retained the same number and chromosomal positions in both lines, and the maize rDNA locus in OMA6.33 was stably maintained without detectable alteration after transfer into the oat genome (Figure 5a, Figure S17). Thus, the alien chromosome is cytologically stable but does not reconfigure the physical organization of the host rDNA system.

We next asked whether the retained maize rDNA locus remains transcriptionally active in the oat nucleus. Because oat and maize IGS regions are strongly diverged in repeat structure, they provide clear species‐specific markers for transcript assignment (Figure S18). Using species‐specific assays, including IGS‐based RT‐PCR, RNA‐seq mapping, and RT‐CAPS targeting coding‐region polymorphisms, we detected only oat‐derived rRNA transcripts and no evidence of maize‐specific expression across multiple tissues (Figure 5b–d, Figure S19; Table S9). Even in OMA6.33, where maize rDNA is physically present, RNA‐seq reads mapped exclusively to the oat reference (Figure 5c). These results showed that stable chromosomal retention of the maize NOR is not sufficient to confer transcriptional competence in the oat background. Instead, the resident oat rDNA array fully dominates rRNA production, while alien maize rDNA is completely silenced.

We then asked whether alien chromosome addition perturbs the transcription of endogenous oat rDNA. SNP‐resolved rDNA variant frequency profiles showed that the oat transcriptional landscape is highly robust to this perturbation (Figure 5e). Variants that were strongly represented in oat genomic DNA and consistently expressed in RNA retained the same pattern in both addition lines (e.g., G3405T and A3424T), indicating stable dominance of specific oat rDNA units. Conversely, variants present in the genome but weakly represented in RNA remained suppressed across all backgrounds (e.g., G5930T and T5931A), pointing to a persistent internal hierarchy of activity within the oat rDNA array. Although a small number of sites showed modest shifts in variant frequency (e.g., C3356A and A4748T), these changes were subtle and did not alter the overall transcriptional architecture. Together, these results revealed a hierarchical mode of rDNA regulation in an interspecific chromosomal context. At the species level, transcription is strictly restricted to the resident oat array, with the alien maize locus remaining fully silent despite stable chromosomal maintenance. At the variant level, endogenous oat rDNA expression is further shaped by a stable internal hierarchy of selective unit usage.

## DISCUSSION

Ribosomal DNA (rDNA) occupies a unique position at the interface of genome organization, epigenetic regulation, and cellular function (Carmo‐Fonseca et al., 2000; Vydzhak et al., 2020). It has long been viewed as a highly repetitive, largely homogeneous, and functionally constrained repeat maintained by concerted evolution (Hall et al., 2022; Long & Dawid, 1980). Our analyses demonstrate that rDNA dosage in oat is highly variable across loci, genotypes, and populations, spanning nearly two orders of magnitude (Figure 1). Growing evidence also indicates that rDNA copy number is highly variable across eukaryotes and has been associated with differences in cellular physiology and genome function (Nelson et al., 2019; Raj et al., 2026; Welfer et al., 2025). In mammals, variation in rDNA copy number has been linked with global transcriptional output, aging, and disease susceptibility (Law et al., 2024; Raj et al., 2026; Rothschild et al., 2024), whereas in plants such as *Arabidopsis* and maize, rDNA dosage has been linked to growth, developmental timing, and stress responses (Delorme‐Hinoux et al., 2023; Li et al., 2018). These observations suggest that rDNA CN is an evolutionarily dynamic genomic feature rather than a simple consequence of genome size or ploidy. Importantly, this variation is not random, but exhibits clear structure associated with population differentiation and subspecies divergence (Parks et al., 2018). One plausible explanation is that elevated rDNA copy number enhances translational capacity or buffering potential under specific developmental, ecological, or domestication‐related conditions (Rodriguez‐Algarra et al., 2024). In hexaploid oat, naked accessions tended to contain more rDNA copies than hulled accessions, but the present data do not distinguish whether this difference reflects domestication, demographic history, population structure, unequal inheritance, or other historical processes (Eickbush & Eickbush, 2007; Lopez et al., 2021; Parks et al., 2019). Functional analyses will therefore be required to determine whether copy‐number differences affect ribosome biogenesis, growth, or environmental responses.

Oat multicopy rDNA arrays also contained extensive sequence polymorphism, with pronounced differences among functional regions of the repeat unit (Figure 2). Most repeat‐copy variation occurred in the IGS and ITS regions, whereas the 18S, 5.8S, and 28S coding regions were more conserved and contained predominantly low‐frequency variants. This uneven distribution of sequence diversity is a recurring feature of rDNA evolution across other eukaryotes (Fultz et al., 2023; Rabanal et al., 2017; Sultanov & Hochwagen, 2022). These patterns indicate that functional constraint and evolutionary flexibility are spatially partitioned within the rDNA repeat unit, and are consistent with stronger evolutionary constraint on rRNA coding sequence than on non‐coding spacers (Parks et al., 2018; Sultanov & Hochwagen, 2022). Nevertheless, several population‐differentiated variants were detected within the 28S rRNA gene (Figure 2), indicating that even highly conserved coding regions can accumulate subtle but reproducible divergence at the population level. Similar intragenomic and interindividual rRNA variants have been reported in mammals and other organisms and have been proposed as a potential source of ribosome heterogeneity (Kim et al., 2021; Kurylo et al., 2018; Parks et al., 2018). Our results therefore support the view that rDNA variation is not simply neutral background noise, but may have functional consequences. One possible implication is that population‐specific rRNA variants in oat contribute to functional differences in ribosome composition or activity under particular developmental or environmental conditions (Genuth & Barna, 2018; Shi et al., 2017; Sims et al., 2021). Furthermore, the presence of co‐varying variants suggests that linked mutations may occur within the same rDNA copies, although short‐read sequencing does not permit reconstruction of complete rDNA haplotypes (Hori et al., 2021; Sims et al., 2019). Long‐read sequencing of intact rDNA units will be needed to resolve the physical linkage of variants and the organization of distinct repeat classes within individual arrays (Rodriguez‐Algarra et al., 2024; Rothschild et al., 2024). Together, these results support a model in which rDNA evolution reflects a dynamic balance between mutation, recombination, and selection, maintaining a structured reservoir of variation within an otherwise highly constrained genomic system (Garcia et al., 2024; Otto et al., 2022; Wang et al., 2023).

Across the *Avena* genus, rDNA variation exhibits a strong phylogenetic signal that closely reflects species relationships and genome composition, consistent with the long‐standing phylogenetic utility of rDNA sequences (Dodsworth et al., 2014; Hillis & Dixon, 1991; Xu et al., 2007). Notably, the most variable regions were not necessarily the most informative for distinguishing among lineages. Although most polymorphisms reside in the IGS, lineage differentiation is driven primarily by variants in the ITS and 28S regions, indicating that evolutionarily informative variation is concentrated in regions under intermediate functional constraint (Figure 3). Repeat‐copy variant frequency distributions also differed among lineages. Many C‐lineage‐specific variants occurred at high frequencies, A‐lineage variants spanned a broader frequency range, and the ACD/CD lineage retained numerous intermediate‐frequency variants in hexaploid accessions. These patterns are consistent with lineage‐specific differences in intragenomic homogenization, but they do not identify the responsible mechanisms. Unequal crossover, gene conversion, changes in array size, demographic history, and differences in the inheritance of parental loci could all contribute to the observed distributions (Hillis et al., 1991; Liao, 2000).

Polyploid genomes often exhibit the coexistence of divergent rDNA variants derived from different subgenomes. Polyploidization was accompanied by pronounced and asymmetric remodeling of parental rDNA composition. C‐lineage‐specific variants were strongly reduced in tetraploid CCDD genomes and remained rare in hexaploid oat, whereas a larger proportion of A‐lineage‐specific variants persisted. Thus, parental rDNA contributions were not retained in a simple additive manner following genome merger. Similar asymmetric retention, reduction, or elimination of parental rDNA arrays has been documented in several allopolyploid systems, including wheat, *Brachypodium*, and other alloploid systems (Borowska‐Zuchowska et al., 2020, 2023; Guo & Han, 2014). Several potential processes may explain this pattern. Biased homogenization during concerted evolution could progressively replace C‐subgenome‐derived repeats through unequal recombination or gene conversion (Hillis et al., 1991; Liao, 2000; Sochorová et al., 2017). Gene conversion is widely recognized as a principal mechanism underlying the concerted evolution of tandemly repeated rDNA arrays (Eickbush & Eickbush, 2007; Parks et al., 2019; Wang et al., 2023). During homologous recombination, gene conversion non‐reciprocally copies sequence information from one repeat unit to another, thereby homogenizing repeat‐unit sequences without altering array size. Repeated rounds of gene conversion can either spread a sequence variant throughout an array or eliminate low‐frequency variants, progressively reducing intragenomic sequence heterogeneity. Acting together with unequal crossover, which primarily changes the copy number and organization of rDNA arrays, these processes can generate the extensive sequence homogenization and lineage‐specific turnover commonly observed in multicopy rDNA families. Alternatively, long‐term transcriptional silencing could promote degeneration, contraction, or eventual loss of inactive arrays (Fultz et al., 2023; Guo & Han, 2014; Tulpová et al., 2021). Differences in the initial dosage, chromosomal organization, or transcriptional competence of parental rDNA loci may also influence their persistence after polyploidization. Epigenetic mechanisms associated with ND, including DNA methylation, histone modification, and chromatin remodeling, could further contribute to the differential maintenance of parental arrays (Borowska‐Zuchowska et al., 2020; Chandrasekhara et al., 2016; Tulpová et al., 2021). However, the present comparative genomic and transcriptomic data do not distinguish among these mechanisms. Future studies combining complete long‐read assemblies with locus‐resolved analyses of individual rDNA arrays will be required to distinguish among these mechanisms. Together, these results support a model in which rDNA evolution in polyploid genomes proceeds through asymmetric and stepwise homogenization. Genome merger initially generates high rDNA diversity, which is subsequently remodeled through biased retention and differential homogenization of subgenome‐specific arrays.

To directly test the strength of ND in an interspecific context, we analyzed oat–maize addition lines (Kynast et al., 2001; Rines et al., 2009). Despite stable chromosomal retention of maize rDNA, no maize‐derived transcripts were detected, whereas endogenous oat rDNA remained fully active (Figure 5). This finding demonstrates strong species‐specific suppression of maize rDNA in the oat genomic background, but it does not identify the underlying mechanism. Similar silencing of alien rDNA has been reported in other hybrid systems, suggesting that transcriptional dominance is primarily controlled by epigenetic mechanisms rather than structural rearrangements (Caperta et al., 2002; Morais‐Cecilio et al., 2000; Preuss & Pikaard, 2007; Silva et al., 2008). Silencing of parental or alien rDNA arrays has been described in diverse hybrids and allopolyploids and is frequently associated with DNA methylation, repressive histone modifications, chromatin compaction, and altered accessibility to the RNA polymerase I transcriptional machinery (Hori et al., 2023). The pronounced divergence in promoter and IGS sequence architecture between oat and maize may further facilitate species‐specific recognition and selective activation of rDNA arrays by host transcriptional factors or Pol I‐associated complexes (Figure S18). Comparative analyses of DNA methylation, chromatin accessibility, histone modifications, Pol I occupancy, and nucleolar association will be required to distinguish stable epigenetic repression from promoter incompatibility or other regulatory constraints.

Within hexaploid oat, the representation of rDNA variants in the rRNA transcript pool differed from their genomic abundance and varied among tissues, developmental stages, and stress treatments (Figure 4). These observations indicate that rRNA variant composition is dynamic and that genomic copy number alone does not fully predict transcript‐level representation. Variant‐level dynamics across development, stress conditions, and OMA lines reveal a second layer of regulation (Figures 4 and 5), in which rDNA usage is quantitatively and reversibly tuned according to cellular context. Nevertheless, the reproducible shifts in variant frequency across biological contexts show that the expressed rRNA pool is compositionally variable rather than invariant. The distinct responses observed among stress treatments further suggest that rRNA variant representation is associated with physiological state, although the causal regulatory mechanisms remain unresolved. Several processes could contribute to this pattern, including differential transcription of rDNA repeat classes, variation in precursor‐rRNA processing or mature‐rRNA stability, and differences in cell‐type composition among samples (Borowska‐Zuchowska et al., 2020; Chandrasekhara et al., 2016; Tulpová et al., 2021). Because short‐read RNA sequencing cannot assign individual variants to complete rDNA repeat units or specific chromosomal loci, the current data do not demonstrate selective activation or repression of particular rDNA copies. Together, these findings support a hierarchical model of rDNA regulation. At the species level, stringent epigenetic control enforces nucleolar dominance and ensures stable rRNA production. At the intragenomic level, flexible and context‐dependent tuning enables selective deployment of rDNA variants. This dual regulatory architecture may allow polyploid genomes to preserve functional stability while retaining regulatory plasticity.

Accumulating evidence across eukaryotes indicates that ribosomes are not uniform entities, but can differ in composition and function, giving rise to the concept of “specialized ribosomes” (Beavan et al., 2025; Harvey et al., 2024; Preiss, 2016; Simsek et al., 2017). Our results extend this framework by suggesting that rDNA sequence variation, coupled with differential transcription, represents an additional source of ribosome heterogeneity. In hexaploid oat, the developmental‐ and stress‐responsive expression of specific rRNA variants indicates that ribosome diversity may arise not only through differences in ribosomal proteins or rRNA modification, but also through differential use of distinct rDNA copies. Such sequence variation could influence rRNA structure, folding, or interactions with translation factors, thereby modulating translational efficiency or specificity (Lindahl, 2024; Liu et al., 2021; Milenkovic & Novoa, 2025; Rodnina, 2016). Polyploidy may further enhance this potential by increasing rDNA copy number and buffering deleterious effects, allowing the retention and selective deployment of sequence variation. However, the present data do not demonstrate the existence of specialized ribosomes or establish that individual rRNA variants alter translation. Long‐read sequencing of full‐length rRNA molecules, locus‐resolved rDNA assemblies, ribosome purification, rRNA modification profiling, ribosome profiling, and functional perturbation of individual variants will be particularly informative. More broadly, the extensive remodeling of rDNA dosage, sequence composition, and transcription across *Avena* illustrates how a multicopy gene family can retain highly conserved cellular functions while undergoing substantial evolutionary change following hybridization and polyploidization. Given the prevalence of polyploidy in plants, this form of regulatory plasticity may represent a general mechanism for balancing cellular robustness with adaptive diversification. Future studies combining ribosome profiling, epigenomic analyses, and functional assays will be critical for linking rDNA variation to ribosome function and phenotypic outcomes.

## EXPERIMENTAL PROCEDURES

### Plant materials

The oat‐maize addition lines (OMA6.33 and OMA9.41), together with their parental lines, hexaploid oat (*Avena sativa* L.) cultivars *Starter I* and *Sun II* and the maize (*Zea mays* L.) inbred line B73, were used in this study. These lines were used for cytological identification and transcriptional analyses to investigate the regulatory characteristics of rDNA expression following alien chromosome addition. These seeds were germinated at room temperature, and subsequently transferred into soil. Plants were grown in a greenhouse under controlled conditions at 22°C with a 16‐h light/8‐h dark photoperiod for 2 weeks prior to sampling.

### Fluorescence in situ hybridization (FISH)

Young root tips (2–3 cm in length) were collected for metaphase chromosome preparation. Fluorescence in situ hybridization (FISH) was performed as previously described (Su et al., 2017). The 45S rDNA oligonucleotide probe was labeled with 5′‐TAMRA, whereas 5S rDNA and subtelomeric probes were labeled with 5′‐FAM. Maize‐specific probes, including centromeric satellite repeats (CentC) and rDNA sequences, were used for FISH assays (Table S10). Fluorescence signals were captured using a fluorescence microscope equipped with a cooled CCD camera and controlled by MetaMorph software. Images were processed using Adobe Photoshop CC 2019.

### Bioinformatics pipeline for sequencing data

Raw WGS and RNA‐seq reads were subjected to quality control using fastp (v0.23.4) to remove adapter sequences, low‐quality bases, and reads shorter than 30 bp (Chen et al., 2018; Li et al., 2018). Clean reads were aligned to the reference rDNA sequences using Bowtie2 (v2.5.2) in end‐to‐end mode (Langmead & Salzberg, 2012). Resulting SAM files were converted to BAM format and sorted using SAMtools (v1.8) (Li et al., 2009). To account for differences in genomic contexts, mapping stringency was adjusted accordingly. A relaxed threshold (MAPQ ≥ 10) was applied for multi‐copy regions such as rDNA, whereas a more stringent threshold (MAPQ ≥ 30) was used for single‐copy regions to ensure unique alignment. Filtered BAM files were used for downstream analyses.

### rDNA copy number estimation

rDNA copy number was estimated using a sequencing depth‐based approach adapted from previous studies (Gibbons et al., 2014; Li et al., 2018). The oat reference genome (OT3098) and corresponding gene annotation files were obtained from the GrainGenes database (https://graingenes.org/GG3/graingenes‐downloads/pepsico‐oat‐ot3098‐v2‐files‐2021). Single‐copy (SC) genomic regions, including introns and exons, were identified based on genome annotation. Candidate sequences were extracted using BEDTools (v2.27) (Quinlan & Hall, 2010) and filtered for uniqueness using BLASTN (v2.9.0; e‐value = 1e−6) (Camacho et al., 2009). Only sequences ≥300 bp with a single genomic hit were retained and further validated by re‐alignment to the reference genome. This process yielded a high‐confidence SC reference set comprising 415 exons and 4049 introns. Sequencing reads were mapped to both the SC reference set, 45S, and 5S rDNA consensus sequence using the pipeline described above. rDNA copy number was calculated as the ratio of mean sequencing depth in rDNA regions to that in SC regions:

$$\text{Copy Number}=\frac{\text{Dept}h_{\text{rDNA}}}{\text{Dept}h_{\mathrm{SC}-\mathrm{EI}}}$$

where $\text{Dept}h_{\text{rDNA}}$ represents the average coverage of the 5S, 18S, 5.8S, 28S, or full‐length regions. $\text{Dept}h_{\mathrm{SC}-\mathrm{EI}}$ was calculated as a trimmed mean (excluding the top and bottom 5% of values) across all SC regions to reduce the influence of outliers and sequencing bias. All statistical analyses and data visualizations were performed in R (v4.0.0) using the tidyverse package (Wickham et al., 2019).

### Quantification of 45S rDNA copy number by qPCR

The relative 45S rDNA copy numbers were estimated by qPCR using primers targeting the 18S region of the 45S rDNA repeat and two single‐copy reference genes (SC1 and SC2). Each sample was analyzed with three technical replicates, and mean Cq values were used for calculation. Relative copy number was calculated using the ΔCq method:

$$\text{Relative copy number}=2^{\left(Cq_{\text{reference}}-Cq_{18S}\right)}$$

Copy number estimates from SC1 and SC2 were calculated independently, and the final value was obtained by averaging ΔCq values from the two reference genes on the log2 scale followed by back‐transformation. Fold changes between cultivars were calculated relative to Starter.

### Read alignment and rDNA variant calling

Variants within the 45S rDNA were identified from genome‐sequencing data of oat adapted from previous studies (Sultanov & Hochwagen, 2022). Per‐base coverage was calculated using BEDTools (v2.27) to assess sequencing depth and uniformity across the rDNA unit (Quinlan & Hall, 2010). Single‐nucleotide polymorphisms (SNPs) and insertions/deletions (InDels) among the multicopy rDNA units were detected using LoFreq (v2.1.5) (Wilm et al., 2012). Prior to variant calling, indel quality scores were incorporated into BAM files using the LoFreq “indelqual” module, followed by rDNA variant detection with the call module. Variants were subjected to filtering steps to improve accuracy. These included removal of annotation lines, extraction of INFO fields, exclusion of homopolymer‐associated variants (HRUN < 4), and application of a minimum rDNA variant frequency threshold (VF > 0.005). For rDNA variants longer than 5 bp, additional base composition filtering was applied, retaining sites with GC‐adjusted proportions < 0.55. Filtered variants were deduplicated and annotated with sample metadata to generate standardized datasets for downstream analyses.

For each polymorphic site in each accession, rDNA variant frequency was calculated as the proportion of sequencing reads supporting the alternative nucleotide among all reads mapped to the corresponding position in the rDNA reference. Because 45S rDNA is organized as multicopy tandem arrays, these values represent the relative abundance of sequence variants among rDNA repeat units within a genome rather than rDNA variant frequencies at independently inherited single‐copy loci. No ancestral‐state inference or outgroup species was used, and the distributions were therefore not constructed as folded or unfolded site frequency spectra.

### Population differentiation and Co‐variation analysis

Differentiation between hulled (OH) and naked (ON) oat populations was assessed at each polymorphic site using the V_st_ index (Redon et al., 2006), where V_t_ represents total variance in rDNA variant frequency (VF), and V_OH_ and V_ON_ represent within‐population variance.

$$V_{\mathrm{st}}=\frac{V_{t}-\frac{V_{s\_\mathrm{OH}}+V_{s\_\mathrm{ON}}}{2}}{V_{t}}$$

For each site, mean VF within populations and the absolute frequency difference ($\mathrm{\Delta VF}=\left|\;{\bar{\mathrm{VF}}}_{\mathrm{OH}}-{\bar{\mathrm{VF}}}_{\mathrm{ON}}\right|$) were calculated. Candidate high‐differentiation sites were defined based on the following criteria: ΔVF within the top 10% or classified as a fully segregated site (absent in one population but present in at least three individuals of the other population); $V_{\mathrm{st}}\geq 0.05$; and VF ≥ 5% in at least one population.

Co‐variation analysis was performed using Pearson correlation based on VF values across samples. Site pairs with strong correlation ($\left|r\right|>0.75$) were retained to identify co‐varying variant clusters within rDNA arrays.

### Phylogenetic and evolutionary dynamics analysis

A sample‐by‐site rDNA variant frequency (VF) matrix was constructed using rDNA polymorphisms across *Avena* lineages. Hierarchical clustering was performed using the R package *ComplexHeatmap* based on Euclidean distance and the Ward.D2 method (Gu et al., 2016). Nonlinear dimensionality reduction was performed using Uniform Manifold Approximation and Projection (UMAP) implemented in the R package *umap* (parameters: n_neighbors = 15, min_dist = 0.1, seed = 123) (McInnes et al., 2018). Two‐dimensional projects were visualized with 95% confidence ellipses using the *stat_ellipse* function (Friendly et al., 2013).

To account for the proportional nature of VF data, values were transformed using an arcsine square‐root transformation prior to downstream statistical analyses (Warton & Hui, 2011). Site‐wise effect size R^2^ was calculated by fitting a linear model (VF ~ lineage), representing the proportion of variance explained by lineage structure. Sites with R^2^ >0.8 were considered highly informative for lineage differentiation.

$$R^{2}=1-\frac{\sum_{i=1}^{n}{\left(y_{i}-\hat{y_{i}}\right)}^{2}}{\sum_{i=1}^{n}{\left(y_{i}-\bar{y}\right)}^{2}}$$

where $y_{i}$ represents the arcsine‐transformed VF value of the sample i, $\hat{y_{i}}$ is the fitted value from the model, and $\bar{y}$ is the mean of the transformed VF values.

To quantify the contribution of different rDNA regions (IGS, 18S, ITS, 5.8S, 28S) to lineage divergence, permutational multivariate analysis of variance (PERMANOVA) was performed using the *adonis2* function in the R package *vegan* with 999 permutations (Oksanen et al., 2012). The proportion of variance explained R^2^ was used to estimate the contribution of each region.

### Sliding‐window analysis of rDNA sequence diversity

Repeat‐copy nucleotide diversity across the 45S rDNA units was calculated based on LoFreq‐derived rDNA variant frequencies. The 9346‐bp repeat sequence was divided into six annotated regions (IGS, 18S, ITS1, 5.8S, ITS2, and 28S). For each polymorphic site, repeat‐copy nucleotide diversity was calculated as 2p(1 − p), where p represents the alternative rDNA variant frequency, and non‐polymorphic sites were assigned a value of zero. A sliding‐window analysis was performed using 100‐bp windows with a 10‐bp step size. Mean nucleotide diversity and variant density were calculated for each window, and accession‐level profiles were summarized to evaluate regional variation patterns across the 45S rDNA repeat.

### Transcriptome‐based rDNA variant analysis

RNA‐seq reads from oat and OMA lines were processed using the same bioinformatics pipeline as genomic sequencing data. Variants were classified as DNA–RNA shared or RNA‐specific sites based on their presence in genomic and transcriptomic datasets. rDNA variant frequency was quantified across tissues, developmental stages, abiotic stress conditions, and OMA backgrounds. Variant distributions and transcriptional patterns were visualized using heatmaps and density plots.

### RNA extraction, RT‐PCR, and RT‐CAPS

Total RNA was extracted from different tissues of oat, maize, and OMA lines using a TRIzol‐based protocol (Chomczynski & Sacchi, 1987). Samples were rapidly frozen in liquid nitrogen and ground into a fine powder using a tissue grinder pre‐cooled with liquid nitrogen. RNA integrity and quality were assessed by 1% agarose gel electrophoresis, and samples were stored at −80°C. RT‐PCR was performed using species‐specific primers designed from divergent regions of the oat and maize rDNA sequences, particularly within the IGS, to distinguish transcript origin.

Two polymorphic SNP sites containing restriction enzyme recognition motifs were selected for cleaved amplified polymorphic sequence (CAPS) assays (Chandrasekhara et al., 2016). Genomic DNA extracted from oat was used as a template. Site‐specific primers were designed to amplify regions flanking each SNP, followed by restriction enzyme digestion. Digested products were resolved by agarose gel electrophoresis to confirm the coexistence of reference and variants within individual genomes.

To assess transcriptional activity and nucleolar dominance in OMA lines, RT‐CAPS was performed on both genomic DNA and complementary DNA (cDNA) (Chandrasekhara et al., 2016). OMA lines and their parental oat and maize lines were included for comparison. Species‐specific SNPs within rDNA were targeted to distinguish parental contributions. Comparison with cDNA digestion patterns revealed the relative transcriptional activity of each parental rDNA. Primer sequences used for RT‐PCR and RT‐CAPS are listed in Table S10.

### Statistical analysis

All statistical analyses were performed in R (v4.0.0) (Team, 2025). Differences in rDNA copy number and rDNA variant frequency between groups were assessed using two‐sided Wilcoxon rank‐sum tests. Population differentiation (Vst) was evaluated using permutation tests (*n* = 5000) and bootstrap resampling (*n* = 2000). Co‐variation among rDNA variant sites was assessed using Pearson correlation, with strongly correlated pairs defined as |r| > 0.75. Unless otherwise specified, statistical significance was defined as *P* < 0.05.

## AUTHOR CONTRIBUTIONS

HDS designed the research; ZYW, HRW, JQ, WTD, XW, and QQG performed the research; ZYW, HRW, and HDS analyzed the data; HDS wrote the article.

## CONFLICT OF INTEREST

The authors declare no competing interests.

## Supporting information


**Figure S1.** Cytological localization of rDNA and validation of sequencing depth in oat. (a) Fluorescence in situ hybridization (FISH) mapping of 45S (red) and 5S (green) rDNA loci in chromosomes of oat (cv. *Sun II*). Chromosomes were counterstained with DAPI (blue). Enlarged panels highlight signals on chromosomes 3D, 4A, and 4D. Scale bar, 20 μm. (b) Box plots showing the sequencing depth distributions for single‐copy gene exons (SCexon) and introns (SCintron). Comparable median depths (~20×) indicate uniform genome‐wide coverage. (c) Scatter plot showing correlation analysis of sequencing depth between SCexon and SCintron regions (r = 0.98, *P* = 7.76 × 10⁻^12^), supporting the reliability of using single‐copy genes for rDNA copy number estimation.
**Figure S2.** Sequencing depth saturation analysis for rDNA copy number estimation across different oat genomes. (a–d) rDNA copy number estimates (CN, × 10^3^) derived from the full‐length 45S rDNA unit (a), 18S (b), 5.8S (c), and 28S (d) regions plotted against sequencing depth (0.5× to 20×). Representative diploids (AlAl: *CN58138* and *CN58139*), tetraploids (CCDD: *CN108634* and *BYU209*), and hexaploids (AACCDD: *Sang* and *Sanfensan*) are shown. Estimates plateau at ~5× coverage, indicating stable and reliable CN estimation across ploidy levels.
**Figure S3.** Distribution of rDNA copy number across oat genomes with different ploidy levels. Box plots compare rDNA copy number (CN, × 10^3^) in diploid (AlAl), tetraploid (CCDD), and hexaploid (AACCDD) genomes. Copy number was independently estimated using the full‐length 45S rDNA unit (orange), 18S (yellow), 5.8S (green), and 28S (blue) regions.
**Figure S4.** Correlation analysis between 5S and 45S rDNA components in hexaploid oat. (a–c) Relationships between 5S rDNA and 18S (a), 5.8S (b), and 28S (c) copy numbers in hexaploid oat populations. Linear regression lines with 95% confidence intervals are shown. Pearson correlation analysis revealed no significant correlations (*P* > 0.05).
**Figure S5.** Characteristics of rDNA variants in hexaploid oat populations. (a) Distribution of indel lengths of rDNA variants detected in oat populations. The histogram shows the number of insertions and deletions (indels) of different sizes. (b) Pie chart summarizes the prevalence of all detected rDNA variants across accessions. Most variants are rare or accession‐specific, whereas a small fraction is shared across all samples.
**Figure S6.** Molecular validation of representative rDNA variants in oat populations. (a) Scatter plots showing the rDNA variant frequency (VF) distributions of two representative variants (left: G3204A; right: C5460T) in hulled oat (OH, red) and naked oat (ON, blue) populations. (b) Experimental validation by cleaved amplified polymorphic sequence (CAPS) assays. The schematic on the left illustrates the strategy for converting sequence variants into restriction enzyme recognition sites. The G3204A substitution creates a *BgI*II recognition site, whereas the C5460T substitution creates a *Sty*I recognition site. On the right, genomic DNA from the oat *Starter I* was amplified by PCR and subjected to restriction digestion. For the G3204A site, both undigested and digested bands were detected, confirming the coexistence of the G variant and the A variant. Similarly, digestion at the C5460T site validated the coexistence of both C and T variants, supporting the heterogeneity of rDNA copies within individual genomes.
**Figure S7.** Distribution of rDNA variant numbers in hexaploid oat and their relationship with rDNA copy number. (a) Density plots summarize the number of variants detected per accession within the full‐length rDNA unit (grey), noncoding regions (IGS + ITS, orange), and coding regions (blue). (b) Sliding‐window analysis depicts the distribution of repeat‐copy nucleotide diversity across the 9346‐bp 45S rDNA unit. Mean nucleotide diversity values were calculated using a 100‐bp window with a 10‐bp step size across 111 hexaploid oat accessions. (c) Sliding‐window analysis shows the distribution of rDNA variant density along the 45S rDNA repeat unit. Shaded regions indicate annotated rDNA subregions, including IGS, 18S, ITS1, 5.8S, ITS2, and 28S. (d) The scatter plot shows the relationship between the number of detected rDNA variants per accession and the corresponding rDNA copy number (× 10^3^).
**Figure S8.** Comparison of rDNA variant abundance and variant frequency distributions between OH and ON populations. (a–c) Violin plots show the total numbers of polymorphic sites detected in hulled oat (OH, *n* = 22) and naked oat (ON, *n* = 89) within the full‐length rDNA unit (a), noncoding regions (IGS + ITS) (b), and coding regions (c). No significant differences were detected between OH and ON in any of the three categories (Wilcoxon rank‐sum test, *P* > 0.05). (d–f) Density plots show the distributions of rDNA variant frequencies for all polymorphic sites across the full‐length rDNA unit (d), noncoding (e), and coding (f) regions.
**Figure S9.** rDNA variant frequency distributions of highly differentiated rDNA variants between hulled and naked oat populations.
Violin plots show the rDNA variant frequency distributions of 40 representative differentiated rDNA variants in OH (red) and ON (blue) populations. For each panel, the genomic position, nucleotide substitution, population differentiation index (Vst), and corresponding rDNA region (IGS, 18S, ITS2, or 28S) are indicated above the plot.
**Figure S10.** Co‐variation map of differentiated rRNA variants in secondary structure models. Localization of differentiated rDNA variants and co‐variation analysis in the secondary structures of 18S, 5.8S, and 28S rRNAs. Red filled circles indicate candidate differentiated variants identified from OH and ON populations, corresponding to the coding‐region sites highlighted in Figure S9. These variants are distributed across multiple structural domains of the rRNAs. Cyan open circles mark the 5′ and 3′ ends of each rRNA molecule. Pearson correlation coefficients were calculated based on rDNA variant frequency matrices across polymorphic sites, and only highly correlated site pairs (|r| > 0.75) were retained. Blue lines connect the eight co‐varying site pairs identified within coding regions.
**Figure S11.** Distribution of rDNA sequence variation across the genus *Avena*.
(a) Box plots showing the abundance of polymorphic sites detected in samples representing 10 distinct genome types. (b) Stacked percentage bar plots show the proportional contributions of the IGS, 18S, ITS, 5.8S, and 28S regions to overall sequence variation in each genome type. (c) Violin plots showing the contribution of single polymorphic sites in different rDNA regions to lineage discrimination. Red labels indicate the numbers of highly informative sites with explanatory power (R^2^) > 0.8 in each region. (d) Bar plots quantify the aggregate explanatory power (overall R^2^) of each rDNA region for lineage partitioning.
**Figure S12.** Prevalence and rDNA variant frequency distributions across major evolutionary lineages in *Avena*. Scatter plots show the distribution of selected rDNA variants in the three major evolutionary lineages of *Avena*. Only variants with prevalence ≥0.2 and rDNA variant frequency ≥5% were retained, as indicated by the red dashed threshold lines. Variants are classified according to their sharing status among lineages as lineage‐specific (Specific), partially shared (Partial‐Shared), or fully shared across all lineages (Fully‐Shared).
**Figure S13.** Asymmetric retention and evolutionary trajectories of A‐ and C‐subgenome‐specific rDNA variants during polyploidization. Heatmaps show the rDNA variant frequency dynamics of diagnostic rDNA variants derived from ancestral diploid lineages across polyploid genomes. Color intensity represents variant frequency. Left, C‐lineage‐specific variants distributed across the IGS, ITS, and 28S regions are shown. Right, A‐lineage‐specific variants located mainly in the IGS and ITS regions are shown.
**Figure S14.** Distribution and rDNA variant frequency dynamics of conserved rDNA variants along the hexapolyploid oat. (a) The schematic shows the positions of representative conserved variants across the rDNA repeat unit. Most variants showing pronounced frequency shifts were concentrated in the 5′ETS, whereas a smaller number were located in ITS1, ITS2, and the 3′ETS region. (b) Bar plots show the rDNA variant frequencies across three genomes (diploid AlAl; allotetraploid CCDD; and allohexaploid AACCDD).
**Figure S15.** Validation of the suitability of transcriptome data for rDNA variant analysis. (a, b) Coverage profiles of rDNA sequences along the full‐length rDNA unit are shown for samples collected at different developmental stages of leaves and panicles (a) and for seedlings subjected to six abiotic stress treatments, including heat, cold, alkali, salt, drought, and flooding (b).
**Figure S16.** Distribution, regional bias, and reliability assessment of transcriptional rDNA variants in hexaploid oat. (a) Scatter plot showing rDNA variant frequencies of variant sites detected consistently across biological replicates within each condition. The x‐axis indicates the physical position along the full‐length 45S rDNA unit, and the y‐axis represents the mean AF across replicates. (b) Stacked bar plots showing the number of variant sites across rDNA regions (IGS, 18S, ITS1, ITS2, and 28S) under different tissues and stress conditions. Variants are categorized as shared with genomic DNA (Shared with DNA) or RNA‐specific (RNA Unique). (c) Scatter plot illustrating the dependence of rDNA variant frequency on sequencing depth (log scale).
**Figure S17.** Cytological localization of alien rDNA in oat–maize addition lines. FISH analysis of chromosomes using maize‐specific rDNA (red) and centromeric CentC (green) in the parental and OMA lines. Enlarged views further confirmed the co‐localization of alien maize chromosomes in oat genome background.
**Figure S18.** Collinearity analysis of oat and maize rDNA sequences and structural divergence in the IGS region. (a) Dot plot comparison of full‐length rDNA sequences between oat and maize. Sequence alignment between oat rDNA (y‐axis) and maize B73 rDNA (x‐axis) reveals strong collinearity across the coding regions. The IGS shows fragmented and discontinuous alignments with multiple short parallel signals, reflecting substantial divergence in sequence composition, length, and tandem repeat organization. (b) Schematic diagrams of the rDNA repeat units are shown, with enlargement of the promoter/enhancer region upstream of the transcription initiation site (TIS). In oat, the IGS contains approximately 3.5 tandem repeat units of ~117 bp each. In maize B73, the corresponding region is composed of a larger and more complex array of approximately 8.5 tandem repeats, each ~200–233 bp in length.
**Figure S19.** Transcriptional analysis of alien rDNA in oat–maize addition lines. Tissue‐specific transcriptional analysis of maize and oat rDNA by RT‐PCR. Species‐specific primers (Maize‐S and Oat‐S) were used to amplify rDNA fragments from genomic DNA and from cDNA derived from root tips, coleoptiles, and leaves.

**Table S1.** rDNA copy number estimates across *Avena* species with different ploidy levels.
**Table S2.** qPCR‐based estimation of 18S rDNA copy number in hexaploid oat cultivars (*Avena sativa* cv. Starter I and Sun II).
**Table S3.** Population‐level variation in rDNA copy number among 111 hexaploid oat accessions.
**Table S4.** Summary of 45S rDNA sequence variants and variant frequencies in 111 hexaploid oat accessions.
**Table S5.** List of candidate high‐differentiation sites between hulled and naked oat.
**Table S6.** Details of 59 *Avena* accessions used for evolutionary and phylogenomic analyses.
**Table S7.** Lineage‐specific 45S rDNA variants among genus *Avena*.
**Table S8.** Summary of RNA‐seq data processing and 45S rDNA alignment quality metrics.
**Table S9.** Transcription of maize‐ and oat‐specific rDNA in oat–maize addition lines.
**Table S10.** Primer and probe sequences used for this study.

## Data Availability

Whole‐genome resequencing (WGS) data from 111 oat accessions were used for rDNA copy number estimation and population‐level variation analyses (BioProject: PRJNA921897) (Nan et al., 2023). To investigate evolutionary relationships, additional WGS datasets from 59 *Avena* accessions were included (BioProject: PRJNA735443, PRJNA556219, PRJNA726919, PRJNA872831, PRJNA838431, PRJCA003205, PRJCA027164, PRJCA030330, and PRJCA016282) (He et al., 2025; Kamal et al., 2022; Li, Liu, et al., 2025; Liu et al., 2020, 2024; Liu, Liu, et al., 2025; Maughan et al., 2019; Nan et al., 2023; Peng et al., 2022). RNA‐seq datasets from oat cv. *Sanfensan* were used to assess transcriptional dynamics of rDNA variants (PRJNA727473) (Peng et al., 2022). Transcriptome data from oat‐maize addition lines were further incorporated to examine rDNA transcription in a heterologous chromosomal context (PRJNA335627) (Dong et al., 2018). Accession numbers for all datasets are provided in Tables S3, S6, S8, and S9.

## References

[tpj71140-bib-0001] Avni, R. , Kamal, N. , Bitz, L. , Jellen, E.N. , Bekele, W.A. , Angessa, T.T. et al. (2025) A pangenome and pantranscriptome of hexaploid oat. Nature, 649, 131–139. 10.1038/s41586-025-09676-7 PMC12727504 41162711

[tpj71140-bib-0002] Bai, X. , Sun, M. , Zhang, Y. , Yin, H. , Cui, X. , Liu, X. et al. (2025) Overcoming breeding constraints in polyploid oat from evolutionary insights. *BioRxiv: The Preprint Server for Biology*, 2025.2007.2018.665467.

[tpj71140-bib-0003] Beavan, A.J.S. , Thuburn, V. , Fatkhullin, B. , Cunningham, J. , Hopes, T.S. , Dimascio, E. et al. (2025) Specialized ribosomes: integrating new insights and current challenges. Philosophical Transactions of the Royal Society of London. Series B, Biological Sciences, 380, 20230377. 10.1098/rstb.2023.0377 PMC11883436 40045788

[tpj71140-bib-0004] Borowska‐Zuchowska, N. , Kovarik, A. , Robaszkiewicz, E. , Tuna, M. , Tuna, G.S. , Gordon, S. et al. (2020) The fate of 35S rRNA genes in the allotetraploid grass Brachypodium hybridum. The Plant Journal, 103, 1810–1825. 10.1111/tpj.14869 PMC7497271 32506573

[tpj71140-bib-0005] Borowska‐Zuchowska, N. , Mykhailyk, S. , Robaszkiewicz, E. , Matysiak, N. , Mielanczyk, L. , Wojnicz, R. et al. (2023) Switch them off or not: selective rRNA gene repression in grasses. Trends in Plant Science, 28, 661–672. 10.1016/j.tplants.2023.01.002 36764871

[tpj71140-bib-0006] Camacho, C. , Coulouris, G. , Avagyan, V. , Ma, N. , Papadopoulos, J. , Bealer, K. et al. (2009) BLAST+: architecture and applications. BMC Bioinformatics, 10, 421. 10.1186/1471-2105-10-421 PMC2803857 20003500

[tpj71140-bib-0007] Caperta, A.D. , Neves, N. , Morais‐Cecílio, L. , Malhó, R. & Viegas, W. (2002) Genome restructuring in rye affects the expression, organization and disposition of homologous rDNA loci. Journal of Cell Science, 115, 2839–2846. 10.1242/jcs.115.14.2839 12082145

[tpj71140-bib-0008] Carmo‐Fonseca, M. , Mendes‐Soares, L. & Campos, I. (2000) To be or not to be in the nucleolus. Nature Cell Biology, 2, E107–E112. 10.1038/35014078 10854340

[tpj71140-bib-0009] Chandrasekhara, C. , Mohannath, G. , Blevins, T. , Pontvianne, F. & Pikaard, C.S. (2016) Chromosome‐specific NOR inactivation explains selective rRNA gene silencing and dosage control in Arabidopsis. Genes & Development, 30, 177–190. 10.1101/gad.273755.115 PMC4719308 26744421

[tpj71140-bib-0010] Chen, J. , Wang, Z. , Tan, K. , Huang, W. , Shi, J. , Li, T. et al. (2023) A complete telomere‐to‐telomere assembly of the maize genome. Nature Genetics, 55, 1221–1231. 10.1038/s41588-023-01419-6 PMC10335936 37322109

[tpj71140-bib-0011] Chen, S. , Zhou, Y. , Chen, Y. & Gu, J. (2018) fastp: an ultra‐fast all‐in‐one FASTQ preprocessor. Bioinformatics, 34, i884–i890. 10.1093/bioinformatics/bty560 PMC6129281 30423086

[tpj71140-bib-0012] Chomczynski, P. & Sacchi, N. (1987) Single‐step method of RNA isolation by acid guanidinium thiocyanate‐phenol‐chloroform extraction. Analytical Biochemistry, 162, 156–159. 10.1006/abio.1987.9999 2440339

[tpj71140-bib-0013] Clemens, R. & van Klinken, B.J. (2014) Oats, more than just a whole grain: an introduction. The British Journal of Nutrition, 112(Suppl 2), S1–S3. 10.1017/s0007114514002712 25405254

[tpj71140-bib-0014] Delorme‐Hinoux, V. , Mbodj, A. , Brando, S. , De Bures, A. , Llauro, C. , Covato, F. et al. (2023) 45S rDNA diversity in natura as one step towards ribosomal heterogeneity in Arabidopsis thaliana. Plants (Basel), 12, 2722. 10.3390/plants12142722 PMC10386311 37514338

[tpj71140-bib-0015] Dodsworth, S. , Chase, M.W. , Kelly, L.J. , Leitch, I.J. , Macas, J. , Novák, P. et al. (2014) Genomic repeat abundances contain phylogenetic signal. Systematic Biology, 64, 112–126. 10.1093/sysbio/syu080 PMC4265144 25261464

[tpj71140-bib-0016] Dong, Z. , Yu, J. , Li, H. , Huang, W. , Xu, L. , Zhao, Y. et al. (2018) Transcriptional and epigenetic adaptation of maize chromosomes in oat‐maize addition lines. Nucleic Acids Research, 46, 5012–5028. 10.1093/nar/gky209 PMC6007749 29579310

[tpj71140-bib-0017] Eickbush, T.H. & Eickbush, D.G. (2007) Finely orchestrated movements: evolution of the ribosomal RNA genes. Genetics, 175, 477–485. 10.1534/genetics.107.071399 PMC1800602 17322354

[tpj71140-bib-0018] Friendly, M. , Monette, G. & Fox, J. (2013) Elliptical insights: understanding statistical methods through elliptical geometry. Statistical Science, 28, 1–39.

[tpj71140-bib-0019] Fultz, D. , McKinlay, A. , Enganti, R. & Pikaard, C. (2023) Sequence and epigenetic landscapes of active and silent nucleolus organizer regions in Arabidopsis. Science Advances, 9, eadj4509. 10.1126/sciadv.adj4509 PMC10619934 37910609

[tpj71140-bib-0020] Garcia, S. , Kovarik, A. , Maiwald, S. , Mann, L. , Schmidt, N. , Pascual‐Díaz, J.P. et al. (2024) The dynamic interplay between ribosomal DNA and transposable elements: a perspective from genomics and cytogenetics. Molecular Biology and Evolution, 41, msae025. 10.1093/molbev/msae025 PMC10946416 38306580

[tpj71140-bib-0021] Genuth, N.R. & Barna, M. (2018) The discovery of ribosome heterogeneity and its implications for gene regulation and organismal life. Molecular Cell, 71, 364–374. 10.1016/j.molcel.2018.07.018 PMC6092941 30075139

[tpj71140-bib-0022] Gibbons, J.G. , Branco, A.T. , Yu, S. & Lemos, B. (2014) Ribosomal DNA copy number is coupled with gene expression variation and mitochondrial abundance in humans. Nature Communications, 5, 4850. 10.1038/ncomms5850 25209200

[tpj71140-bib-0023] Gu, Z. , Eils, R. & Schlesner, M. (2016) Complex heatmaps reveal patterns and correlations in multidimensional genomic data. Bioinformatics, 32, 2847–2849. 10.1093/bioinformatics/btw313 27207943

[tpj71140-bib-0024] Guo, X. & Han, F. (2014) Asymmetric epigenetic modification and elimination of rDNA sequences by polyploidization in wheat. The Plant Cell, 26, 4311–4327. 10.1105/tpc.114.129841 PMC4277213 25415973

[tpj71140-bib-0025] Hall, A.N. , Morton, E. & Queitsch, C. (2022) First discovered, long out of sight, finally visible: ribosomal DNA. Trends in Genetics, 38, 587–597. 10.1016/j.tig.2022.02.005 PMC10132741 35272860

[tpj71140-bib-0026] Handa, H. , Kanamori, H. , Tanaka, T. , Murata, K. , Kobayashi, F. , Robinson, S.J. et al. (2018) Structural features of two major nucleolar organizer regions (NORs), nor‐B1 and nor‐B2, and chromosome‐specific rRNA gene expression in wheat. The Plant Journal, 96, 1148–1159. 10.1111/tpj.14094 30238531

[tpj71140-bib-0027] Harvey, R.F. , Pöyry, T. & Willis, A.E. (2024) Let's (P‐s)talk about specialized ribosomes. Molecular Cell, 84, 4478–4479. 10.1016/j.molcel.2024.11.011 39642853

[tpj71140-bib-0028] He, Q. , Xiao, Y. , Li, T. , Wang, Y. , Wang, Y. , Wang, Y. et al. (2025) High‐quality genome of allotetraploid *Avena barbata* provides insights into the origin and evolution of B subgenome in *Avena* . Journal of Integrative Plant Biology, 67, 1515–1532. 10.1111/jipb.13902 PMC12131679 40226959

[tpj71140-bib-0029] Hillis, D.M. & Dixon, M.T. (1991) Ribosomal DNA: molecular evolution and phylogenetic inference. The Quarterly Review of Biology, 66, 411–453. 10.1086/417338 1784710

[tpj71140-bib-0030] Hillis, D.M. , Moritz, C. , Porter, C.A. & Baker, R.J. (1991) Evidence for biased gene conversion in concerted evolution of ribosomal DNA. Science, 251, 308–310. 10.1126/science.1987647 1987647

[tpj71140-bib-0031] Hori, Y. , Engel, C. & Kobayashi, T. (2023) Regulation of ribosomal RNA gene copy number, transcription and nucleolus organization in eukaryotes. Nature Reviews. Molecular Cell Biology, 24, 414–429. 10.1038/s41580-022-00573-9 36732602

[tpj71140-bib-0032] Hori, Y. , Shimamoto, A. & Kobayashi, T. (2021) The human ribosomal DNA array is composed of highly homogenized tandem clusters. Genome Research, 31, 1971–1982. 10.1101/gr.275838.121 PMC8559705 34407983

[tpj71140-bib-0033] Hotz, M. , Thayer, N.H. , Hendrickson, D.G. , Schinski, E.L. , Xu, J. & Gottschling, D.E. (2022) rDNA array length is a major determinant of replicative lifespan in budding yeast. Proceedings of the National Academy of Sciences of the United States of America, 119, e2119593119. 10.1073/pnas.2119593119 PMC9169770 35394872

[tpj71140-bib-0034] Kamal, N. , Tsardakas Renhuldt, N. , Bentzer, J. , Gundlach, H. , Haberer, G. , Juhasz, A. et al. (2022) The mosaic oat genome gives insights into a uniquely healthy cereal crop. Nature, 606, 113–119. 10.1038/s41586-022-04732-y PMC9159951 35585233

[tpj71140-bib-0035] Kim, J.‐H. , Noskov, V.N. , Ogurtsov, A.Y. , Nagaraja, R. , Petrov, N. , Liskovykh, M. et al. (2021) The genomic structure of a human chromosome 22 nucleolar organizer region determined by TAR cloning. Scientific Reports, 11, 2997. 10.1038/s41598-021-82565-x PMC7862453 33542373

[tpj71140-bib-0036] Kurylo, C.M. , Parks, M.M. , Juette, M.F. , Zinshteyn, B. , Altman, R.B. , Thibado, J.K. et al. (2018) Endogenous rRNA sequence variation can regulate stress response gene expression and phenotype. Cell Reports, 25, 236–248.e236. 10.1016/j.celrep.2018.08.093 PMC6312700 30282032

[tpj71140-bib-0037] Kynast, R.G. , Riera‐Lizarazu, O. , Vales, M.I. , Okagaki, R.J. , Maquieira, S.B. , Chen, G. et al. (2001) A complete set of maize individual chromosome additions to the oat genome. Plant Physiology, 125, 1216–1227. 10.1104/pp.125.3.1216 PMC65602 11244103

[tpj71140-bib-0038] Langmead, B. & Salzberg, S.L. (2012) Fast gapped‐read alignment with Bowtie 2. Nature Methods, 9, 357–359. 10.1038/nmeth.1923 PMC3322381 22388286

[tpj71140-bib-0039] Law, P.P. , Mikheeva, L.A. , Rodriguez‐Algarra, F. , Asenius, F. , Gregori, M. , Seaborne, R.A.E. et al. (2024) Ribosomal DNA copy number is associated with body mass in humans and other mammals. Nature Communications, 15, 5006. 10.1038/s41467-024-49397-5 PMC11169392 38866738

[tpj71140-bib-0040] Lee, S. , Seo, Y.‐E. , Choi, J. , Yan, X. , Kim, T. , Choi, D. et al. (2024) Nucleolar actions in plant development and stress responses. Plant, Cell and Environment, 47, 5189–5204. 10.1111/pce.15099 39169813

[tpj71140-bib-0041] Leitch, A.R. & Leitch, I.J. (2008) Genomic plasticity and the diversity of polyploid plants. Science, 320, 481–483. 10.1126/science.1153585 18436776

[tpj71140-bib-0042] Li, B. , Kremling, K.A.G. , Wu, P. , Bukowski, R. , Romay, M.C. , Xie, E. et al. (2018) Coregulation of ribosomal RNA with hundreds of genes contributes to phenotypic variation. Genome Research, 28, 1555–1565. 10.1101/gr.229716.117 PMC6169892 30166407

[tpj71140-bib-0043] Li, H. , Handsaker, B. , Wysoker, A. , Fennell, T. , Ruan, J. , Homer, N. et al. (2009) The sequence alignment/map format and SAMtools. Bioinformatics, 25, 2078–2079. 10.1093/bioinformatics/btp352 PMC2723002 19505943

[tpj71140-bib-0044] Li, W. , Liu, J. , Liu, N. , Liang, H. , Zhang, L. , Sun, Q. et al. (2025) Genomic insights into the divergence between hulled and hulless oats. Cell Reports, 44, 116055. 10.1016/j.celrep.2025.116055 40711879

[tpj71140-bib-0045] Li, W. , Wang, Y. , Liu, J. , He, Q. , Zhou, Y. , Li, M. et al. (2025) A gap‐free complete genome assembly of oat and OatOmics, a multi‐omics database. Molecular Plant, 18, 179–182. 10.1016/j.molp.2025.01.006 39780493

[tpj71140-bib-0046] Liao, D. (2000) Gene conversion drives within genic sequences: concerted evolution of ribosomal RNA genes in bacteria and archaea. Journal of Molecular Evolution, 51, 305–317. 10.1007/s002390010093 11040282

[tpj71140-bib-0047] Lindahl, L. (2024) Ribosome structural changes dynamically affect ribosome function. International Journal of Molecular Sciences, 25, 11186. 10.3390/ijms252011186 PMC11508205 39456968

[tpj71140-bib-0048] Liu, F. , Bratulić, S. , Costello, A. , Miettinen, T.P. & Badran, A.H. (2021) Directed evolution of rRNA improves translation kinetics and recombinant protein yield. Nature Communications, 12, 5638. 10.1038/s41467-021-25852-5 PMC8463689 34561441

[tpj71140-bib-0049] Liu, J. , Liu, N. , Yan, W. , Hu, X. , Wang, M. , Qin, R. et al. (2025) Reference genome and population genomic analyses reveal insight into herbicide tolerance in *Avena fatua* L. Nature Communications, 16, 9851. 10.1038/s41467-025-64825-w PMC12594974 41203602

[tpj71140-bib-0050] Liu, Q. , Li, X. , Li, M. , Xu, W. , Schwarzacher, T. & Heslop‐Harrison, J.S. (2020) Comparative chloroplast genome analyses of *Avena*: insights into evolutionary dynamics and phylogeny. BMC Plant Biology, 20, 406. 10.1186/s12870-020-02621-y PMC7466839 32878602

[tpj71140-bib-0051] Liu, Q. , Xiong, G. , Wang, Z. , Wu, Y. , Tu, T. , Schwarzacher, T. et al. (2024) Chromosome‐level genome assembly of the diploid oat species *Avena longiglumis* . Sci Data, 11, 412. 10.1038/s41597-024-03248-6 PMC11035610 38649380

[tpj71140-bib-0052] Liu, Q. , Yi, C. , Zhang, Z. , Su, H. , Liu, C. , Huang, Y. et al. (2023) Non‐B‐form DNA tends to form in centromeric regions and has undergone changes in polyploid oat subgenomes. Proceedings of the National Academy of Sciences of the United States of America, 120, e2211683120. 10.1073/pnas.2211683120 PMC9910436 36574697

[tpj71140-bib-0053] Liu, S. , Li, K. , Dai, X. , Qin, G. , Lu, D. , Gao, Z. et al. (2025) A telomere‐to‐telomere genome assembly coupled with multi‐omic data provides insights into the evolution of hexaploid bread wheat. Nature Genetics, 57, 1008–1020. 10.1038/s41588-025-02137-x PMC11985340 40195562

[tpj71140-bib-0054] Long, E.O. & Dawid, I.B. (1980) Repeated genes in eukaryotes. Annual Review of Biochemistry, 49, 727–764. 10.1146/annurev.bi.49.070180.003455 6996571

[tpj71140-bib-0055] Lopez, F.B. , Fort, A. , Tadini, L. , Probst, A.V. , McHale, M. , Friel, J. et al. (2021) Gene dosage compensation of rRNA transcript levels in Arabidopsis thaliana lines with reduced ribosomal gene copy number. The Plant Cell, 33, 1135–1150. 10.1093/plcell/koab020 PMC8225240 33793816

[tpj71140-bib-0056] Maughan, P.J. , Lee, R. , Walstead, R. , Vickerstaff, R.J. , Fogarty, M.C. , Brouwer, C.R. et al. (2019) Genomic insights from the first chromosome‐scale assemblies of oat (*Avena* spp.) diploid species. BMC Biology, 17, 92. 10.1186/s12915-019-0712-y PMC6874827 31757219

[tpj71140-bib-0057] McInnes, L. , Healy, J. , Saul, N. & Großberger, L. (2018) UMAP: uniform manifold approximation and projection. The Journal of Open Source Software, 3, 861.

[tpj71140-bib-0058] Milenkovic, I. & Novoa, E.M. (2025) Dynamic rRNA modifications as a source of ribosome heterogeneity. Trends in Cell Biology, 35, 604–614. 10.1016/j.tcb.2024.10.001 39500673

[tpj71140-bib-0059] Morais‐Cecilio, L. , Delgado, M. , Jones, R.N. & Viegas, W. (2000) Modification of wheat rDNA loci by rye B chromosomes: a chromatin organization model. Chromosome Research, 8, 341–351. 10.1023/a:1009291714371 10919725

[tpj71140-bib-0060] Nan, J. , Ling, Y. , An, J. , Wang, T. , Chai, M. , Fu, J. et al. (2023) Genome resequencing reveals independent domestication and breeding improvement of naked oat. GigaScience, 12, giad061. 10.1093/gigascience/giad061 PMC10390318 37524540

[tpj71140-bib-0061] Navashin, M. (1934) Chromosome alterations caused by hybridization and their bearing upon certain general genetic problems. Cytologia, 5, 169–203.

[tpj71140-bib-0062] Nelson, J.O. , Watase, G.J. , Warsinger‐Pepe, N. & Yamashita, Y.M. (2019) Mechanisms of rDNA copy number maintenance. Trends in Genetics, 35, 734–742. 10.1016/j.tig.2019.07.006 PMC6744303 31395390

[tpj71140-bib-0063] Oksanen, J. , Blanchet, F.G. , Kindt, R. , Legendre, P. , Minchin, P.R. , O'Hara, R.B. et al. (2012) Vegan: community ecology package. Softw. http://CRAN.R‐project.org/package=vegan

[tpj71140-bib-0064] Otto, M. , Zheng, Y. & Wiehe, T. (2022) Recombination, selection, and the evolution of tandem gene arrays. Genetics, 221, iyac052. 10.1093/genetics/iyac052 PMC9252282 35460227

[tpj71140-bib-0065] Otto, S.P. (2007) The evolutionary consequences of polyploidy. Cell, 131, 452–462. 10.1016/j.cell.2007.10.022 17981114

[tpj71140-bib-0066] Parks, M. , Kurylo, C. , Dass, R. , Bojmar, L. , Lyden, D. , Vincent, C. et al. (2018) Variant ribosomal RNA alleles are conserved and exhibit tissue‐specific expression. Science Advances, 4, eaao0665. 10.1126/sciadv.aao0665 PMC5829973 29503865

[tpj71140-bib-0067] Parks, M.M. , Kurylo, C.M. , Batchelder, J.E. , Theresa Vincent, C. & Blanchard, S.C. (2019) Implications of sequence variation on the evolution of rRNA. Chromosome Research, 27, 89–93. 10.1007/s10577-018-09602-w PMC6505490 30719681

[tpj71140-bib-0068] Peng, Y. , Yan, H. , Guo, L. , Deng, C. , Wang, C. , Wang, Y. et al. (2022) Reference genome assemblies reveal the origin and evolution of allohexaploid oat. Nature Genetics, 54, 1248–1258. 10.1038/s41588-022-01127-7 PMC9355876 35851189

[tpj71140-bib-0069] Preiss, T. (2016) All ribosomes are created equal. really? Trends in Biochemical Sciences, 41, 121–123. 10.1016/j.tibs.2015.11.009 26682497

[tpj71140-bib-0070] Preuss, S. & Pikaard, C.S. (2007) rRNA gene silencing and nucleolar dominance: insights into a chromosome‐scale epigenetic on/off switch. Biochimica et Biophysica Acta, 1769, 383–392. 10.1016/j.bbaexp.2007.02.005 PMC2000449 17439825

[tpj71140-bib-0071] Quinlan, A.R. & Hall, I.M. (2010) BEDTools: a flexible suite of utilities for comparing genomic features. Bioinformatics, 26, 841–842. 10.1093/bioinformatics/btq033 PMC2832824 20110278

[tpj71140-bib-0072] R Core Team . (2025) R: A language and environment for statistical computing. Vienna, Austria: R Foundation for Statistical Computing. https://www.r‐project.org/.

[tpj71140-bib-0073] Rabanal, F.A. , Mandáková, T. , Soto‐Jiménez, L.M. , Greenhalgh, R. , Parrott, D.L. , Lutzmayer, S. et al. (2017) Epistatic and allelic interactions control expression of ribosomal RNA gene clusters in Arabidopsis thaliana. Genome Biology, 18, 75. 10.1186/s13059-017-1209-z PMC5414317 28464948

[tpj71140-bib-0074] Raj, A. , Brown, J.S. , Thayer, N.H. , Hotz, M. , Lam, I. , Fong, N. et al. (2026) Ribosomal DNA copy number variation shapes human physiology and disease risk. *medRxiv*, 2026.2001.2009.26343685.

[tpj71140-bib-0075] Redon, R. , Ishikawa, S. , Fitch, K.R. , Feuk, L. , Perry, G.H. , Andrews, T.D. et al. (2006) Global variation in copy number in the human genome. Nature, 444, 444–454. 10.1038/nature05329 PMC2669898 17122850

[tpj71140-bib-0076] Riera‐Lizarazu, O. , Rines, H.W. & Phillips, R.L. (1996) Cytological and molecular characterization of oat x maize partial hybrids. Theoretical and Applied Genetics, 93, 123–135. 10.1007/BF00225737 24162209

[tpj71140-bib-0077] Rines, H.W. , Phillips, R.L. , Kynast, R.G. , Okagaki, R.J. , Galatowitsch, M.W. , Huettl, P.A. et al. (2009) Addition of individual chromosomes of maize inbreds B73 and Mo17 to oat cultivars Starter and Sun II: maize chromosome retention, transmission, and plant phenotype. Theoretical and Applied Genetics, 119, 1255–1264. 10.1007/s00122-009-1130-2 19707741

[tpj71140-bib-0078] Rodnina, M.V. (2016) The ribosome in action: tuning of translational efficiency and protein folding. Protein Science, 25, 1390–1406. 10.1002/pro.2950 PMC4972197 27198711

[tpj71140-bib-0079] Rodriguez‐Algarra, F. , Evans, D.M. & Rakyan, V.K. (2024) Ribosomal DNA copy number variation associates with hematological profiles and renal function in the UK Biobank. Cell Genomics, 4, 100562. 10.1016/j.xgen.2024.100562 PMC11228893 38749448

[tpj71140-bib-0080] Rothschild, D. , Susanto, T.T. , Sui, X. , Spence, J.P. , Rangan, R. , Genuth, N.R. et al. (2024) Diversity of ribosomes at the level of rRNA variation associated with human health and disease. Cell Genomics, 4, 100629. 10.1016/j.xgen.2024.100629 PMC11480859 39111318

[tpj71140-bib-0081] Sáez‐Vásquez, J. & Delseny, M. (2019) Ribosome biogenesis in plants: from functional 45S ribosomal DNA organization to ribosome assembly factors. The Plant Cell, 31, 1945–1967. 10.1105/tpc.18.00874 PMC6751116 31239391

[tpj71140-bib-0082] Shi, Z. , Fujii, K. , Kovary, K.M. , Genuth, N.R. , Röst, H.L. , Teruel, M.N. et al. (2017) Heterogeneous ribosomes preferentially translate distinct subpools of mRNAs genome‐wide. Molecular Cell, 67, 71–83.e77. 10.1016/j.molcel.2017.05.021 PMC5548184 28625553

[tpj71140-bib-0083] Silva, M. , Pereira, H.S. , Bento, M. , Santos, A.P. , Shaw, P. , Delgado, M. et al. (2008) Interplay of ribosomal DNA loci in nucleolar dominance: dominant NORs are up‐regulated by chromatin dynamics in the wheat‐rye system. PLoS One, 3, e3824. 10.1371/journal.pone.0003824 PMC2585015 19048103

[tpj71140-bib-0084] Sims, J. , Copenhaver, G.P. & Schlogelhofer, P. (2019) Meiotic DNA repair in the nucleolus employs a nonhomologous end‐joining mechanism. The Plant Cell, 31, 2259–2275. 10.1105/tpc.19.00367 PMC6751124 31266898

[tpj71140-bib-0085] Sims, J. , Sestini, G. , Elgert, C. , von Haeseler, A. & Schlogelhofer, P. (2021) Sequencing of the Arabidopsis NOR2 reveals its distinct organization and tissue‐specific rRNA ribosomal variants. Nature Communications, 12, 387. 10.1038/s41467-020-20728-6 PMC7810690 33452254

[tpj71140-bib-0086] Simsek, D. , Tiu, G.C. , Flynn, R.A. , Byeon, G.W. , Leppek, K. , Xu, A.F. et al. (2017) The mammalian Ribo‐interactome reveals ribosome functional diversity and heterogeneity. Cell, 169, 1051–1065.e1018. 10.1016/j.cell.2017.05.022 PMC5548193 28575669

[tpj71140-bib-0087] Sochorová, J. , Coriton, O. , Kuderová, A. , Lunerová, J. , Chèvre, A.‐M. & Kovařík, A. (2017) Gene conversion events and variable degree of homogenization of rDNA loci in cultivars of Brassica napus. Annals of Botany, 119, 13–26. 10.1093/aob/mcw187 PMC5218374 27707747

[tpj71140-bib-0088] Su, H. , Liu, Y. , Dong, Q. , Feng, C. , Zhang, J. , Liu, Y. et al. (2017) Dynamic location changes of Bub1‐phosphorylated‐H2AThr133 with CENH3 nucleosome in maize centromeric regions. The New Phytologist, 214, 682–694. 10.1111/nph.14415 28079247

[tpj71140-bib-0089] Sultanov, D. & Hochwagen, A. (2022) Varying strength of selection contributes to the intragenomic diversity of rRNA genes. Nature Communications, 13, 7245. 10.1038/s41467-022-34989-w PMC9700816 36434003

[tpj71140-bib-0090] Tucker, S. , Vitins, A. & Pikaard, C.S. (2010) Nucleolar dominance and ribosomal RNA gene silencing. Current Opinion in Cell Biology, 22, 351–356. 10.1016/j.ceb.2010.03.009 PMC2912983 20392622

[tpj71140-bib-0091] Tulpová, Z. , Kovařík, A. , Toegelová, H. , Navrátilová, P. , Kapustová, V. , Hřibová, E. et al. (2021) Anatomy, transcription dynamics and evolution of wheat ribosomal RNA loci deciphered by a multi‐omics approach. *bioRxiv: The Preprint Server for Biology*. 10.1002/tpg2.20191 PMC12806993 35092350

[tpj71140-bib-0092] Vydzhak, O. , Luke, B. & Schindler, N. (2020) Non‐coding RNAs at the eukaryotic rDNA locus: RNA–DNA hybrids and beyond. Journal of Molecular Biology, 432, 4287–4304. 10.1016/j.jmb.2020.05.011 32446803

[tpj71140-bib-0093] Wang, K. , Wu, Y. , Zhang, W. , Dawe, R.K. & Jiang, J. (2014) Maize centromeres expand and adopt a uniform size in the genetic background of oat. Genome Research, 24, 107–116. 10.1101/gr.160887.113 PMC3875851 24100079

[tpj71140-bib-0094] Wang, W. , Zhang, X. , Garcia, S. , Leitch, A.R. & Kovařík, A. (2023) Intragenomic rDNA variation ‐ the product of concerted evolution, mutation, or something in between? Heredity, 131, 179–188. 10.1038/s41437-023-00634-5 PMC10462631 37402824

[tpj71140-bib-0095] Warton, D.I. & Hui, F.K.C. (2011) The arcsine is asinine: the analysis of proportions in ecology. Ecology, 92, 3–10. 10.1890/10-0340.1 21560670

[tpj71140-bib-0096] Welfer, G.A. , Brady, R.A. , Natchiar, S.K. , Watson, Z.L. , Rundlet, E.J. , Alejo, J.L. et al. (2025) Impacts of ribosomal RNA sequence variation on gene expression and phenotype. Philosophical Transactions of the Royal Society of London. Series B, Biological Sciences, 380, 20230379. 10.1098/rstb.2023.0379 PMC11883441 40045785

[tpj71140-bib-0097] Wickham, H. , Averick, M. , Bryan, J. , Chang, W. , McGowan, L. , François, R. et al. (2019) Welcome to the Tidyverse. The Journal of Open Source Software, 4, 1686.

[tpj71140-bib-0098] Wilm, A. , Aw, P.P. , Bertrand, D. , Yeo, G.H. , Ong, S.H. , Wong, C.H. et al. (2012) LoFreq: a sequence‐quality aware, ultra‐sensitive variant caller for uncovering cell‐population heterogeneity from high‐throughput sequencing datasets. Nucleic Acids Research, 40, 11189–11201. 10.1093/nar/gks918 PMC3526318 23066108

[tpj71140-bib-0099] Xu, P. , Han, Y. , Wu, J. , Lv, H. , Qiu, L. , Chang, R. et al. (2007) Phylogenetic analysis of the sequences of rDNA internal transcribed spacer (ITS) of *Phytophthora sojae* . Journal of Genetics and Genomics, 34, 180–188. 10.1016/S1673-8527(07)60019-8 17469790

[tpj71140-bib-0100] Zhang, H. , Liu, N. , Wang, Y. , Zheng, X. , Li, W. , Liu, Z. et al. (2025) Super‐pangenome analyses across 35 accessions of 23 *Avena* species highlight their complex evolutionary history and extensive genomic diversity. Nature Genetics, 57, 2276–2288. 10.1038/s41588-025-02294-z 40835889

