# Supplementary material for: Dynamic evolution and hierarchical regulation of rDNA in polyploid oat
Source: Plant J. 2026 Sep 22;127(6):e71140. doi: 10.1111/tpj.71140 (PMC13595461; doi:10.1111/tpj.71140)
Supplement: Supplementary file 1 [file TPJ-127-0-s001.docx]

**Fig. S1 Cytological localization of rDNA and validation of sequencing depth in oat.**

**(a)** Fluorescence in situ hybridization (FISH) mapping of 45S (red) and 5S (green) rDNA loci in chromosomes of oat (cv. *Sun II*). Chromosomes were counterstained with DAPI (blue). Enlarged panels highlight signals on chromosomes 3D, 4A, and 4D. Scale bar, 20 μm. **(b)** Box plots showing the sequencing depth distributions for single-copy gene exons (SCexon) and introns (SCintron). Comparable median depths (~20×) indicate uniform genome-wide coverage. **(c)** Scatter plot showing correlation analysis of sequencing depth between SCexon and SCintron regions (r = 0.98, P = 7.76 × 10⁻¹²), supporting the reliability of using single-copy genes for rDNA copy number estimation.

**Fig. S2 Sequencing depth saturation analysis for rDNA copy number estimation across different oat genomes.**

**(a–d)** rDNA copy number estimates (CN, × 10³) derived from the full-length 45S rDNA unit **(a)**, 18S **(b)**, 5.8S **(c)**, and 28S **(d)** regions plotted against sequencing depth (0.5× to 20×). Representative diploids (AlAl: *CN58138* and *CN58139*), tetraploids (CCDD: *CN108634* and *BYU209*), and hexaploids (AACCDD: *Sang* and *Sanfensan*) are shown. Estimates plateau at ~5× coverage, indicating stable and reliable CN estimation across ploidy levels.

**Fig. S3 Distribution of rDNA copy number across oat genomes with different ploidy levels.**

Box plots compare rDNA copy number (CN, × 10³) in diploid (AlAl), tetraploid (CCDD), and hexaploid (AACCDD) genomes. Copy number was independently estimated using the full-length 45S rDNA unit (orange), 18S (yellow), 5.8S (green), and 28S (blue) regions.

**Fig. S4 Correlation analysis between 5S and 45S rDNA components in hexaploid oat.**

**(a–c)** Relationships between 5S rDNA and 18S **(a)**, 5.8S **(b)**, and 28S **(c)** copy numbers in hexaploid oat populations. Linear regression lines with 95% confidence intervals are shown. Pearson correlation analysis revealed no significant correlations (*P* > 0.05).

**Fig. S5 Characteristics of rDNA variants in hexaploid oat populations.**
**(a)** Distribution of indel lengths of rDNA variants detected in oat populations. The histogram shows the number of insertions and deletions (indels) of different sizes. **(b)** Pie chart summarizes the prevalence of all detected rDNA variants across accessions. Most variants are rare or accession-specific, whereas a small fraction is shared across all samples.

**Fig. S6 Molecular validation of representative rDNA variants in oat populations.**
**(a)** Scatter plots showing the rDNA variant frequency (VF) distributions of two representative variants (left: G3204A; right: C5460T) in hulled oat (OH, red) and naked oat (ON, blue) populations. **(b)** Experimental validation by cleaved amplified polymorphic sequence (CAPS) assays. The schematic on the left illustrates the strategy for converting sequence variants into restriction enzyme recognition sites. The G3204A substitution creates a BgIII recognition site, whereas the C5460T substitution creates a StyI recognition site. On the right, genomic DNA from the oat Starter I was amplified by PCR and subjected to restriction digestion. For the G3204A site, both undigested and digested bands were detected, confirming the coexistence of the G variant and the A variant. Similarly, digestion at the C5460T site validated the coexistence of both C and T variants, supporting the heterogeneity of rDNA copies within individual genome.

**Fig. S7 Distribution of rDNA variant numbers in hexaploid oat and their relationship with rDNA copy number.**
**(a)** Density plots summarize the number of variants detected per accession within the full-length rDNA unit (gray), noncoding regions (IGS + ITS, orange), and coding regions (blue). **(b)** Sliding-window analysis depicts the distribution of repeat-copy nucleotide diversity across the 9,346-bp 45S rDNA unit. Mean **nucleotide diversity** values were calculated using a 100-bp window with a 10-bp step size across 111 hexaploid oat accessions. **(c)** Sliding-window analysis shows the distribution of rDNA variant density along the 45S rDNA repeat unit. Shaded regions indicate annotated rDNA subregions, including IGS, 18S, ITS1, 5.8S, ITS2, and 28S. **(d)** The scatter plot shows the relationship between the number of detected rDNA variants per accession and the corresponding rDNA copy number (× 10³).

**Fig. S8 Comparison of rDNA variant abundance and variant frequency distributions between OH and ON populations.**

**(a–c)** Violin plots show the total numbers of polymorphic sites detected in hulled oat (OH, n = 22) and naked oat (ON, n = 89) within the full-length rDNA unit **(a)**, noncoding regions (IGS + ITS) **(b)**, and coding regions **(c)**. No significant differences were detected between OH and ON in any of the three categories (Wilcoxon rank-sum test, P > 0.05). **(d–f)** Density plots show the distributions of rDNA variant frequencies for all polymorphic sites across the full-length rDNA unit **(d)**, noncoding **(e)**, and coding **(f)** regions.

**Fig. S9 rDNA variant frequency distributions of highly differentiated rDNA variants between hulled and naked oat populations.**

Violin plots show the rDNA variant frequency distributions of 40 representative differentiated rDNA variants in OH (red) and ON (blue) populations. For each panel, the genomic position, nucleotide substitution, population differentiation index (Vst), and corresponding rDNA region (IGS, 18S, ITS2, or 28S) are indicated above the plot.

**Fig. S10 Co-variation map of differentiated rRNA variants in secondary structure models.**

Localization of differentiated rDNA variants and co-variation analysis in the secondary structures of 18S, 5.8S, and 28S rRNAs. Red filled circles indicate candidate differentiated variants identified from OH and ON populations, corresponding to the coding-region sites highlighted in Fig S9. These variants are distributed across multiple structural domains of the rRNAs. Cyan open circles mark the 5’ and 3’ ends of each rRNA molecule. Pearson correlation coefficients were calculated based on rDNA variant frequency matrices across polymorphic sites, and only highly correlated site pairs (|r| > 0.75) were retained. Blue lines connect the eight co-varying site pairs identified within coding regions.

**Fig. S11 Distribution of rDNA sequence variation across the genus *Avena*.**

**(a)** Box plots showing the abundance of polymorphic sites detected in samples representing 10 distinct genome types. **(b)** Stacked percentage bar plots show the proportional contributions of the IGS, 18S, ITS, 5.8S, and 28S regions to overall sequence variation in each genome type. **(c)** Violin plots showing the contribution of single polymorphic sites in different rDNA regions to lineage discrimination. Red labels indicate the numbers of highly informative sites with explanatory power (R²) > 0.8 in each region. **(d)** Bar plots quantify the aggregate explanatory power (overall R²) of each rDNA region for lineage partitioning.

**Fig. S12 Prevalence and rDNA variant frequency distributions across major evolutionary lineages in *Avena*.**

Scatter plots show the distribution of selected rDNA variants in the three major evolutionary lineages of Avena. Only variants with prevalence ≥ 0.2 and rDNA variant frequency ≥ 5% were retained, as indicated by the red dashed threshold lines. Variants are classified according to their sharing status among lineages as lineage-specific (Specific), partially shared (Partial-Shared), or fully shared across all lineages (Fully-Shared).

**Fig. S13 Asymmetric retention and evolutionary trajectories of A- and C-subgenome-specific rDNA variants during polyploidization.**

Heatmaps show the rDNA variant frequency dynamics of diagnostic rDNA variants derived from ancestral diploid lineages across polyploid genomes. Color intensity represents variant frequency. Left, C-lineage-specific variants distributed across the IGS, ITS, and 28S regions are shown. Right, A-lineage-specific variants located mainly in the IGS and ITS regions are shown.

**Fig. S14 Distribution and rDNA variant frequency dynamics of conserved rDNA variants along the hexapolyploid oat.**
**(a)** The schematic shows the positions of representative conserved variants across the rDNA repeat unit. Most variants showing pronounced frequency shifts were concentrated in the 5’ETS), whereas a smaller number were located in ITS1, ITS2, and the 3’ETS region. **(b)** Bar plots show the rDNA variant frequencies across three genomes (diploid AlAl; allotetraploid CCDD; and allohexaploid AACCDD).

**Fig. S15 Validation of the suitability of transcriptome data for rDNA variant analysis.**
**(a–b)** Coverage profiles of rDNA sequences along the full-length rDNA unit are shown for samples collected at different developmental stages of leaves and panicles **(a)** and for seedlings subjected to six abiotic stress treatments, including heat, cold, alkali, salt, drought, and flooding **(b)**.

**Fig. S16 Distribution, regional bias, and reliability assessment of transcriptional rDNA variants in hexaploid oat.**

**(a) Scatter plot showing** rDNA variant **frequencies of variant sites detected consistently across biological replicates within each condition. The x-axis indicates the physical position along the full-length 45S rDNA unit, and the y-axis represents the mean AF across replicates. (b) Stacked bar plots showing the number of variant sites across rDNA regions (IGS, 18S, ITS1, ITS2, and 28S) under different tissues and stress conditions. Variants are categorized as shared with genomic DNA (Shared with DNA) or RNA-specific (RNA Unique). (c) Scatter plot illustrating the dependence of rDNA variant frequency on sequencing depth (log scale).**

**Fig. S17 Cytological localization of alien rDNA in oat–maize addition lines.**
FISH analysis of chromosomes using maize specific rDNA (red) and centromeric CentC (green) in the parental and OMA lines. Enlarged views further confirmed the co-localization of alien maize chromosomes in oat genome background.

**Fig. S18 Collinearity analysis of oat and maize rDNA sequences and structural divergence in the IGS region.**
**(a)** Dot plot comparison of full-length rDNA sequences between oat and maize. Sequence alignment between oat rDNA (y-axis) and maize B73 rDNA (x-axis) reveals strong collinearity across the coding regions. The IGS shows fragmented and discontinuous alignments with multiple short parallel signals, reflecting substantial divergence in sequence composition, length, and tandem repeat organization. **(b)** Schematic diagrams of the rDNA repeat units are shown, with enlargement of the promoter/enhancer region upstream of the transcription initiation site (TIS). In oat, the IGS contains approximately 3.5 tandem repeat units of ~117 bp each. In maize B73, the corresponding region is composed of a larger and more complex array of approximately 8.5 tandem repeats, each ~200–233 bp in length.

**Fig. S19 Transcriptional analysis of alien rDNA in oat–maize addition lines.**
Tissue-specific transcriptional analysis of maize and oat rDNA by RT-PCR. Species-specific primers (Maize-S and Oat-S) were used to amplify rDNA fragments from genomic DNA and from cDNA derived from root tips, coleoptiles, and leaves.
